# Manifold-adaptive dimension estimation revisited

**DOI:** 10.7717/peerj-cs.790

**Published:** 2022-01-06

**Authors:** Zsigmond Benkő, Marcell Stippinger, Roberta Rehus, Attila Bencze, Dániel Fabó, Boglárka Hajnal, Loránd G. Eröss, András Telcs, Zoltán Somogyvári

**Affiliations:** 1 Department of Computational Sciences, Wigner Research Centre for Physics, Budapest, Hungary; 2 János Szentágothai Doctoral School of Neurosciences, Semmelweis University, Budapest, Hungary; 3 Epilepsy Center, Department of Neurology, National Institute of Clinical Neurosciences, Budapest, Hungary; 4 Department of Functional Neurosurgery, National Institute of Clinical Neurosciences, Budapest, Hungary; 5 Faculty of Information Technology and Bionics, Péter Pázmány Catholic University, Budapest, Hungary; 6 Department of Computer Science and Information Theory, Faculty of Electrical Engineering and Informatics, Budapest University of Technology and Economics, Budapest, Hungary; 7 Department of Quantitative Methods, Faculty of Business and Economics,, University of Pannonia, Veszprém, Hungary; 8 Neuromicrosystems ltd., Budapest, Hungary

**Keywords:** Fractal dimension, Intrinsic dimension, Epilepsy, EEG, Dynamical systems, Manifold, DANCo, Takens theorem, Maximum likelihood, Causality

## Abstract

Data dimensionality informs us about data complexity and sets limit on the structure of successful signal processing pipelines. In this work we revisit and improve the manifold adaptive Farahmand-Szepesvári-Audibert (FSA) dimension estimator, making it one of the best nearest neighbor-based dimension estimators available. We compute the probability density function of local FSA estimates, if the local manifold density is uniform. Based on the probability density function, we propose to use the median of local estimates as a basic global measure of intrinsic dimensionality, and we demonstrate the advantages of this asymptotically unbiased estimator over the previously proposed statistics: the mode and the mean. Additionally, from the probability density function, we derive the maximum likelihood formula for global intrinsic dimensionality, if i.i.d. holds. We tackle edge and finite-sample effects with an exponential correction formula, calibrated on hypercube datasets. We compare the performance of the corrected median-FSA estimator with kNN estimators: maximum likelihood (Levina-Bickel), the 2NN and two implementations of DANCo (R and MATLAB). We show that corrected median-FSA estimator beats the maximum likelihood estimator and it is on equal footing with DANCo for standard synthetic benchmarks according to mean percentage error and error rate metrics. With the median-FSA algorithm, we reveal diverse changes in the neural dynamics while resting state and during epileptic seizures. We identify brain areas with lower-dimensional dynamics that are possible causal sources and candidates for being seizure onset zones.

## Background

Dimensionality sets profound limits on the stage where data takes place, therefore it is often crucial to know the intrinsic dimension of data to carry out meaningful analysis. Intrinsic dimension provides direct information about data complexity; as such, it was recognised as a useful measure to describe the dynamics of dynamical systems (Grassberger & Procaccia, 1983), to detect anomalies in time series (Houle, Schubert & Zimek, 2018), to diagnose patients with various conditions (Dlask & Kukal, 2017; Polychronaki et al., 2010; Sharma, Pachori & Rajendra Acharya, 2017; Acharya et al., 2013) and to use it simply as plugin parameter for signal processing algorithms.

Most of the multivariate datasets lie on a lower dimensional manifold embedded in a potentially very high-dimensional embedding space. This is because the observed variables are far from independent, and this interdependence introduces redundancies resulting in a lower intrinsic dimension (ID) of data compared with the number of observed variables. To capture this—possibly nonlinear—interdependence, nonlinear dimension-estimation techniques can be applied to reveal connections between the variables in the dataset (Sugiyama & Borgwardt, 2013; Romano et al., 2016), particularly between time series (Benkő et al., 2018; Krakovská, 2019). In this latter case, the estimated intrinsic dimension provides actionable information about the causal structures within the investigated system based on it’s dynamics.

Dimension estimation of system’s dynamics from time series is supported by theorems of nonlinear dynamical systems. Given a univariate time series generated by a deterministic chaotic dynamical system one can reconstruct the multivariate state of the system, for example, by time delay embedding if some mild conditions are met (Packard et al., 1980; Takens, 1981). This procedure is carried out by adding the time shifted versions of the time series to itself as new coordinates:

(1) \begin{eqnarray*}X(t)=[x(t),x(t-\tau ),x(t-2\tau ),\ldots ,x(t-(E-1)\tau ),x(t-(E-1)\tau )]\end{eqnarray*}

where *x*(*t*) is the time series, *X*(*t*) is the reconstructed state. *E* and *τ* are two parameters, the embedding dimension and embedding delay respectively.

State space reconstruction by time delay embedding or some other technique based on wavelet transformation (Parlitz & Mayer-Kress, 1995; You & Huang, 2011; Hu et al., 2019) or recurrent neural networks (Chen et al., 2018; de Brouwer et al., 2019) are usually a first step in any nonlinear time series analysis pipeline to characterize the system’s dynamics (Bradley & Kantz, 2015). In the E-dimensional embedding space, the intrinsic dimensionality of the augmented dataset can be a relevant real-time descriptor of the dynamics (Skinner, Molnar & Tomberg, 1994).

To estimate the ID of data various approaches have been proposed, for a full review of techniques see the work of Campadelli et al. (2015). Here we discuss the k-Nearest Neighbor (kNN) ID estimators, with some recent advancements in the focus.

A usually basic assumption of *k*NN ID estimators is that the fraction of points *f* in a spherical neighborhood is approximately determined by the intrinsic dimensionality (*D*) and radius (*R*) times a—locally almost constant—mostly density-dependent factor (*η*(*x*, *R*), Eq. (2)).

(2) \begin{eqnarray*}f\approx \eta (x,R){R}^{D}\end{eqnarray*}

where *f* is the fraction of samples in a neighborhood.

Assuming the Poisson sampling process on the manifold, Levina & Bickel (2004) derived a Maximum Likelihood estimator, which became a popular method and got several updates (Ghahramani & Mckay, 2005; Gupta & Huang, 2010). These estimators are prone to underestimation of dimensionality because of finite sample effects and overestimations because of the curvature.

To address the challenges posed by curvature and finite sample, new estimators were proposed (Rozza et al., 2012; Bassis et al., 2015; Ceruti et al., 2014; Facco et al., 2017). To tackle the effect of curvature, a minimal neighborhood size can be taken on normalized neighborhood distances as in the case of MIND_ML_ (Rozza et al., 2012). To tackle the underestimation due to finite sample effects, empirical corrections were applied. A naive empirical correction approach was applied by Camastra & Vinciarelli (2002): a perceptron was trained on the estimates computed for randomly sampled hypercubes to learn a correction function. Motivated by the correction in the previous work, the IDEA method was created (Rozza et al., 2012); and a more principled approach was carried out, where the full distribution of estimates was compared to the distributions computed on test data sets using the Kullback–Leibler divergence (MIND_KL_ (Rozza et al., 2012), DANCo (Ceruti et al., 2014)). In the case of DANCo, not just the nearest neighbor distances, but the angles are measured and taken into account in the estimation process resulting in more accurate estimates.

In the recent years, further estimators have been proposed, such as the estimator that uses minimal neighborhood information leveraging the empirical distribution of the ratio of the nearest neighbors to fit intrinsic dimension (Facco et al., 2017), or other approaches based on simplex skewness (Johnsson, Soneson & Fontes, 2015) and normalized distances (Chelly, Houle & Kawarabayashi, 2016; Amsaleg et al., 2015; Amsaleg et al., 2018; Amsaleg et al., 2019).

**Figure 1:**
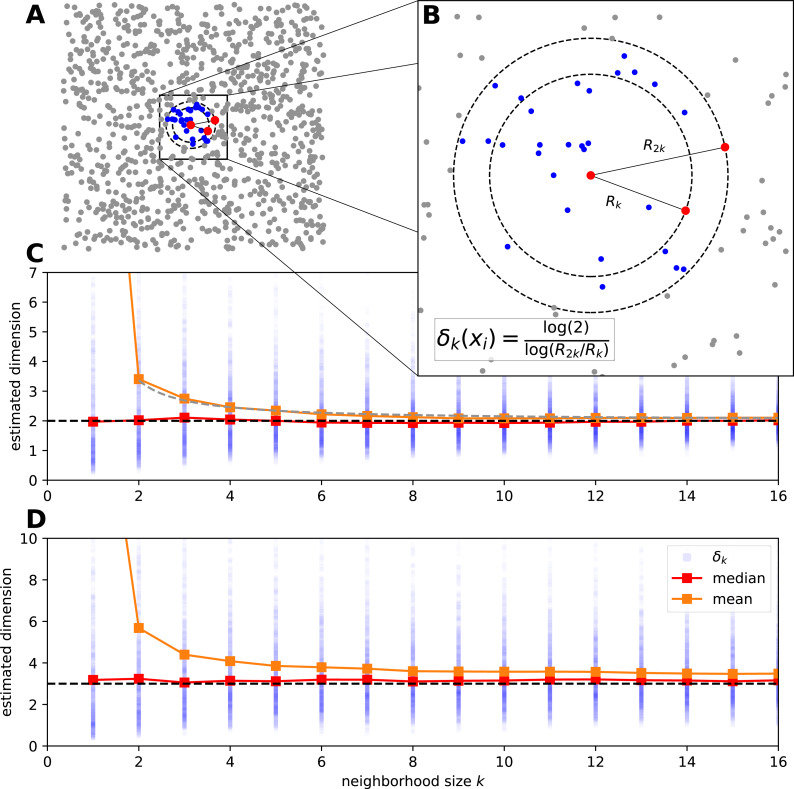
The estimation procedure of manifold-adaptive Farahmand-Szepesvári-Audibert intrinsic dimension estimator. (A) The data is a set of uniformly sampled points from the [0, 1] × [0, 1] interval (*n* = 10^3^). A neighborhood around the most central sample point is colored by blue. (B) A magnified view shows the neighborhood around the central sample point. The local FSA estimate (*δ*_*k*_(*x*_*i*_)) is computed leveraging the formula for the distances of the *k*th and 2*k*th neighbor. This computation is repeated for the whole sample and a global estimate is generated as the mean of the local estimates. (C) We show the local estimates (blue dots), the empirical mean (orange) and median (red) in the function of a neighborhood size for the 2D points above. The mean has an upcurving tail at small neigborhood sizes but the median seems to be robust global estimate even for the smallest neighborhood. The mean approximately lies on a hyperbola $ \frac{aD}{k-1} +D\approx \lt {\delta }_{k}({x}_{i}){\gt }_{i}$, where *a* ≈ 0.685 is a constant (grey dashed line). (D) We measure the intrinsic dimension of the dynamics for a logistic map driven by two other independent logistic maps (*n* = 1, 000). We show the local FSA estimates (blue), the mean (orange) and the median (red) in the function of neighborhood size after time delay embedding (*E* = 4, *τ* = 1). The dynamics is approximately 3 dimensional and the median robustly reflects this, however the mean overestimates the intrinsic dimension at small neigborhood sizes.

In the followings we revisit the manifold-adaptive Farahmand-Szepesvári-Audibert (FSA) dimension estimator proposed by Farahmand, Szepesvári & Audibert (2007) to measure intrinsic dimensionality of datasets (Fig. 1). This estimator is extremely simple, it uses two neighborhoods around a data point to estimate the local intrinsic dimensionality.

We derive the FSA estimator from Eq. (2). Let $\mathcal{M}$ be a *D* dimensional manifold and let’s have a sample {*x*_*i*_} where *i* ∈ {1, 2, …, *n*} with size n, sampled from $\mathcal{M}$. We take two neigborhoods around a sample point, thereby we fix *f* = *k*/*n* and if ${R}_{k}^{i}$ is the distance at which the *k*-th neighbor is found around *x*_*i*_, then we can take the logarithm of both sides:

(3) \begin{eqnarray*}\ln \nolimits \left( \frac{k}{n} \right) & \approx \ln \nolimits ~\eta +D~\ln \nolimits {R}_{k}^{i}\ln \nolimits \left( \frac{2k}{n} \right) & \approx \ln \nolimits ~{\eta }^{{^{\prime}}}+D~\ln \nolimits {R}_{2k}^{i}\end{eqnarray*}

If *η* is slowly varying and Δ*R* is small, we can take *η* = *η*′ as a constant. Thus, by subtracting the two equations from each other we get rid of the local density dependence:

(4) \begin{eqnarray*}\ln \nolimits \left( 2 \right) \approx D~\ln \nolimits \left( \frac{{R}_{2k}^{i}}{{R}_{k}^{i}} \right) \end{eqnarray*}

We rearrange Eq. (4) to compute the local estimates, which is practically fitting a line through the log-distance of the *k*th and 2*k*th nearest neighbor at a given sample’s location (Figs. 1A, 1B):

(5) \begin{eqnarray*}{\delta }_{k}({x}_{i})= \frac{\ln \nolimits (2)}{\ln \nolimits \left( {R}_{2k}^{i}/{R}_{k}^{i} \right) } \end{eqnarray*}

where *δ*_*k*_(*x*_*i*_) is the local FSA dimension estimate.

To compute a global ID estimate, the authors proposed the mean of local estimates at sample-points, or a vote for the winner global ID value (the mode), if the estimator is used in integer-mode. They proved that the above global ID estimates are consistent for *k* > 1, if *η* is differentiable and the manifold is regular. They calculated the upper bound for the probability of error for the global estimate, however this bound contains unknown constants (Farahmand, Szepesvári & Audibert, 2007).

In practice one computes the local estimates for various neighborhood sizes and compute the global estimate typically by averaging. We show this procedure by two examples: on uniformly sampled points from the 2D plane and on a coupled logistic map system (Figs. 1C, 1D). For the uniform random sample the basic assumptions of the FSA method hold, and the average of local values estimates well the global dimension *D* = 2 at bigger neighborhood sizes (*k* > 8). However for small neighborhood sizes the estimate curls upwards and goes to infinity at *k* = 1 (Fig. 1C). One can use a robust statistic, the median as a global estimate and gets better results.

As a second example let’s see the intrinsic dimension estimation procedure for a coupled logistic map system to grasp the complexity of the system’s dynamics. We couple three chaotic logistic maps, such that two independent variables drive a third one through nonlinear coupling:

(6) \begin{eqnarray*}x(t+1)={r}_{x}x(1-x)y(t+1)={r}_{y}y(1-y)z(t+1)={r}_{z}z(1-z-{\beta }_{zx}x-{\beta }_{zy}y)\end{eqnarray*}

where *x*, *y* and *z* ∈ [0, 1] are the state variables, *r*_*i*_ = 3.99 and *β*_*i*_ = 0.3 are parameters. We generate *n* = 10^3^ sample points with periodic boundary on the [0, 1] interval and investigate the dynamics of the variable *z*. We apply time delay embedding with embedding dimension *E* = 4 and embedding delay *τ* = 1, and compute the local FSA estimates around each sample in the embedding space with periodic boundary conditions (Fig. 1D). At small neighborhoods the mean of the local estimates is higher than the actual intrinsic dimensionality (*D* ≈ 3) of the data, the median however stays approximately constant with respect to *k*.

We showed in the previous two examples that the median of local FSA estimates was a more robust estimator of the intrinsic dimension than the mean, but the generality of this finding is yet to be explored by more rigorous means. Additionally, in these cases the data were abundant, and the edge effect was softened by periodic boundary, but data can be scarce and the manifold may have finite size causing systematic errors in the estimates of intrinsic dimension.

In this paper we propose an improved FSA estimator, based on the assumption that the density is locally uniform. The main contributions of this paper are as follows:

(1) We calculate the probability density function of local FSA estimates, and derive formula for the sampling distribution of the median.
(2) We prove that the median is an asymptotically unbiased estimate of the intrinsic dimension, and introduce this variant as the median FSA (mFSA) algorithm. To confirm the validity of the theory, we make comparison with empirical measurements carried out on uniformly sampled random hypercube datasets with varied sample size and intrinsic dimension value. We find that finite sample size and edge effects cause systematic underestimation at high intrinsic dimensions.
(3) We present the new corrected median FSA (cmFSA) method to alleviate the underestimation due to finite sample and edge effects. We achieve this by applying a heuristic exponential correction-formula applied on the mFSA estimate and we test the new algorithm on benchmark datasets.
(4) Finally, we apply the mFSA estimator to locate putative epileptic focus on Local Field Potential measurements of a human subject.

The paper is organised as follows. In the Methods section, we present the steps of FSA, mFSA and cmFSA algorithms, then we describe the simulation of the hypercube datasets and we show the specific calibration procedure used in the cmFSA method. After these, we turn to benchmark datasets. We refer to data generation scripts and display the evaluation procedure. This section ends with a description of Local Field Potential measurements and the analysis workflow. In the Results section, we lay out the theoretical results about the FSA estimator first, then we validate them against simple simulations as second. Third, we compare our algorithms on benchmark datasets against standard methods. Fourth, we apply the mFSA algorithm on Local Field Potential measurements. These parts are followed by the Discussion and Conclusion sections.

## Methods

### The FSA and mFSA algorithm

There is a dataset with a sample size *n*, and sample points *x*_*i*_ ∈ ℝ^*m*^. Then,

1. **Compute distances:** Calculate the distance of the *k*th and 2*k*th nearest neighbors (*R*_*k*_, *R*_2*k*_) for each data point (*x*_*i*_). Here the neighborhood size is some positive integer *k* ∈ ℤ^+^.
2. **Compute local estimates:** Get local estimates *δ*_*k*_(*x*_*i*_) from the distances for each data point according to Eq. (5).
3. **Calculate global estimate:** Aggregate the local estimates into one global value. This last step is the only difference between the FSA and the mFSA method:
  (a) FSA estimator:
    (7) \begin{eqnarray*}{d}_{FSA}^{(k)}= \frac{\sum {\delta }_{k}({x}_{i})}{n} \end{eqnarray*}
  (b) mFSA estimator:
    (8) \begin{eqnarray*}{d}_{mFSA}^{(k)}=\text{M}[\{ {\delta }_{k}({x}_{1}),{\delta }_{k}({x}_{2}),\ldots ,{\delta }_{k}({x}_{n})\} ]\end{eqnarray*}
    where M stands for the sample median.

### The cmFSA algorithm

There is a dataset with a sample size *n*, and sample points *x*_*i*_ ∈ ℝ^*m*^. Then,

1. **Compute mFSA estimate** Apply the mFSA algorithm to get biased global estimate ${d}_{mFSA}^{(k)}$.
2. **Model Calibration** Fit a correction-model with the the given sample size *n* on uniform random hypercube calibration datasets consisting of various intrinsic dimension values, many instances each (at least *N* = 15 realizations). We used the following model:
  (9) \begin{eqnarray*}D\approx d\exp \nolimits \left( \sum _{l=1}^{L}{\alpha }_{l}{d}^{l} \right) \end{eqnarray*}
  where *D* is the true dimension of the underlying manifold, *α*_*l*_-s are sample size and *k* dependent coefficients, *L* is the order of the polynomial and $d={d}_{mFSA}^{(k)}$ is a shorthand for the biased local estimate. This model is derived from heuristic reasoning, and simplifies to a linear model in the parameters, if the logarithm of the two sides is taken. First we calculate biased estimates on each test data. Second, we carry out the model fit by linear regression on the log–log values with the ordinary least squares method or with orthogonal distance regression.
3. **Calculate cmFSA estimate** Plug in the biased estimate into fitted the correction model to compute ${d}_{cmFSA}^{(k)}$.

A python implementation of the algorithms can be found at https://github.com/phrenico/cmfsapy along with the supporting codes for this article.

### Simulations on D-hypercube datasets

The simulations were implemented in python3 (Van Rossum & Drake, 2009) using the numpy (Oliphant, 2006), scipy (Virtanen et al., 2020) and matplotlib (Hunter, 2007) packages, unless otherwise stated.

We generated test-datasets by uniform random sampling from the unit *D*-cube to demonstrate, that theoretical derivations fit to data. We measured distances with a circular boundary condition to avoid edge effects, hence the data is as close to the theoretical assumptions as possible.

**Figure 2:**
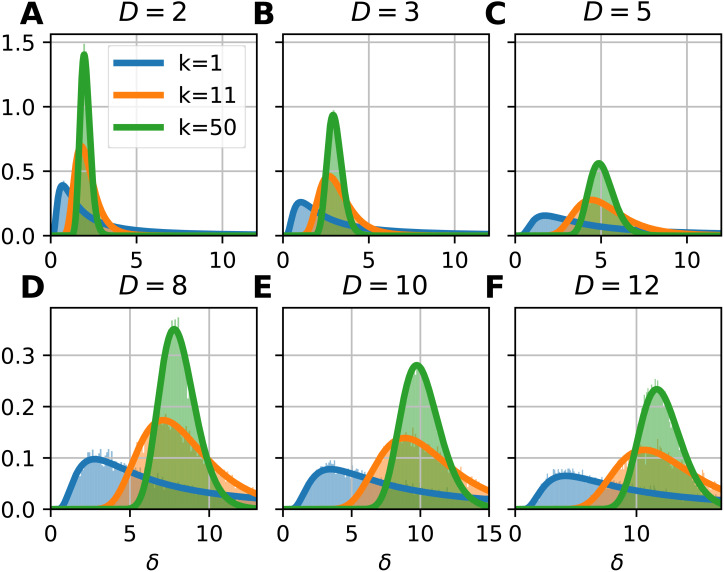
Probability density functions of the local Farahmand-Szepesvári-Audibert estimator (*δ*) for various dimensions (*D*) and neighborhood sizes (*k*). (A–F) The sublots show that the theoretical probability density functions (pdfs) (continuous lines) fit to the histograms (*n* = 10, 000) of local estimates calculated on uniformly sampled hypercubes (*D* = 2, 3, 5, 8, 10, 12). The three colors denote the three presented neigborhood sizes: *k* = 1 (blue), *k* = 11 (orange) and *k* = 50 (green). The pdfs are less skewed and the variance gets smaller as the neighborhood size gets bigger. Also, the higher the dimension of the manifold, the higher the variance of the local estimates.

To illustrate the probability density function of the FSA estimator, we computed the local FSA intrinsic dimension values (Fig. 2). We generated *d*-hypercubes (*n* = 10, 000, one realization) with dimensions of 2, 3, 5, 8, 10 and 12, then computed histograms of local FSA estimates for three neighborhood sizes: *k* = 1, 11, 50 respectively (Figs. 2A, 2F). We selected these specific neighborhoods because of didactic purposes: the *k* = 1 neighborhood is the smallest one, the *k* = 50 is a bigger neighborhood, which is still much smaller than the sample size, so the estimates are not affected by the finite sample effect. The *k* = 11 neighborhood represents a transition between the two “extremes”, the specific value is an arbitrary choice giving pleasing visuals suggesting the gradual change in the shape of the curve as a function of the *k* parameter. We drew the theoretically computed probability density function (pdf) to illustrate the fit.

**Figure 3:**
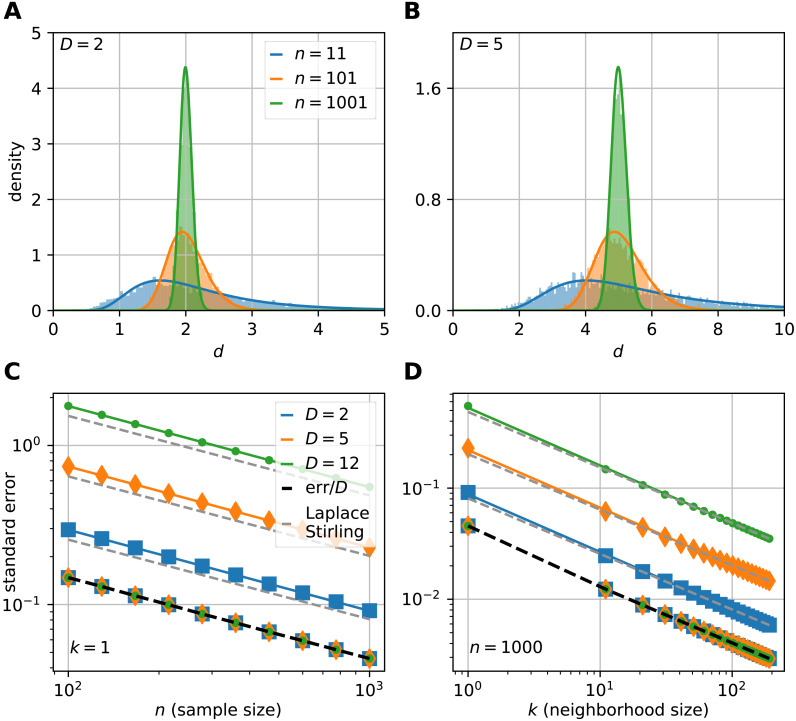
The sampling distribution and standard error of the median for the FSA estimator on uniformly sampled hypercubes. The figure shows the pdf of median-FSA estimator of points uniformly sampled from two example systems: a square (A) and from a 5D hypercube (B) for three sample sizes *n* = 11 (blue), *n* = 101 (orange) and *n* = 1, 001 (green) respectively for the smallest neighborhood (*k* = 1). The solid lines represent the theoretical pdfs of the median and the shaded histograms are the results of simulations (*N* = 5, 000 realizations of hypercube datasets with periodic boundary conditions). The derived formula fits well to the histograms. The variance shrinks with bigger sample size, and the pdf becomes less skewed, more Gaussian-like. (C) The standard error of median in the function of sample size computed by numerical integration and Laplace-Stirling approximation (grey dashed). The standard error linearly decreases on a log–log plot in the function of sample size. The slope is approximately −0.5, independent of the dimension of the manifold and the error’s value is proportional to *D*. Thus, the relative error (*err*/*D*) is independent of intrinsic dimension and it is shown by the overlapping markers on the black dashed straight line. (D) The standard error in the function of neighborhood size computed by numerical integration and Laplace-Stirling approximation. The slope of the lines are also approximately −0.5, the apprximation (grey dashed line) becomes accurate for *k* > 10 neighborhood size.

To show that the theoretically computed sampling distribution of the mFSA fits to the hypercube datasets, we varied the sample size (*n* = 11, 101, 1001) with *N* = 5, 000 realizations from each. We computed the global mFSA for each realization and plotted the results for *d* = 2 (Fig. 3A) and *d* = 5 (Fig. 3B).

**Figure 4:**
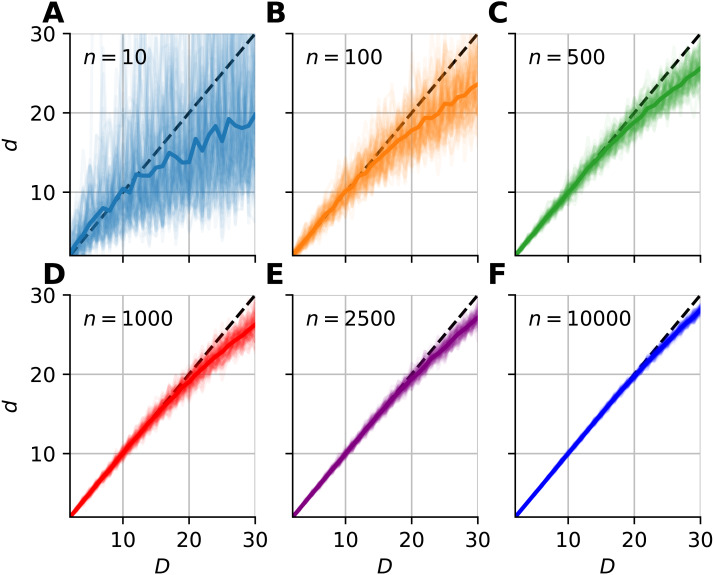
Intrinsic dimension dependence of the median-FSA estimator for uniformly sampled unit hypercubes with various sample sizes (*k* = 1) with periodic boundary conditions. Subplots (A–F) show the mean of median-FSA estimator (thick line) values from *N* = 100 realizations (shading) of uniformly sampled unit hypercubes . The perfect estimation values lie on the diagonal (dashed black line). As the intrinsic dimension of the manifold grows, the estimates start to deviate from the ideal diagonal line due to finite sample effect. This systematic under-estimation of intrinsic dimension is more severe in the case of low sample size and high intrinsic dimension.

**Figure 5:**
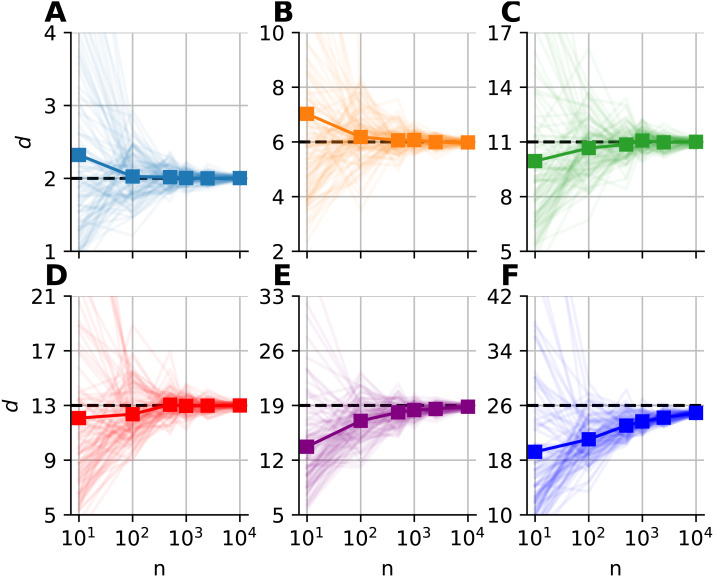
Sample size dependence of the median-FSA estimator for uniformly sampled unit hypercubes with varied intrinsic dimension value and peiodic boundary (*k* = 1). Subplots (A–F) show the mean of median-FSA estimator (thick line) values from *N* = 100 realizations (shading). The estimator asymptotically converges to the true dimension value, but the convergence is faster for lower intrinsic dimensions.

We investigated the dimensionality and sample-size effects on mFSA estimates (Figs. 4–5). We simulated the hypercube data in the 2–30 dimension-range, and applied various sample sizes: *n* = 10, 100, 1, 000, 2, 500, 10, 000; one hundred realizations each (*N* = 100). We computed the mFSA values with minimal neighborhood size (*k* = 1), and observed finite-sample-effects, and asymptotic convergence. We repeated the analysis with hard boundary conditions.

**Figure 6:**
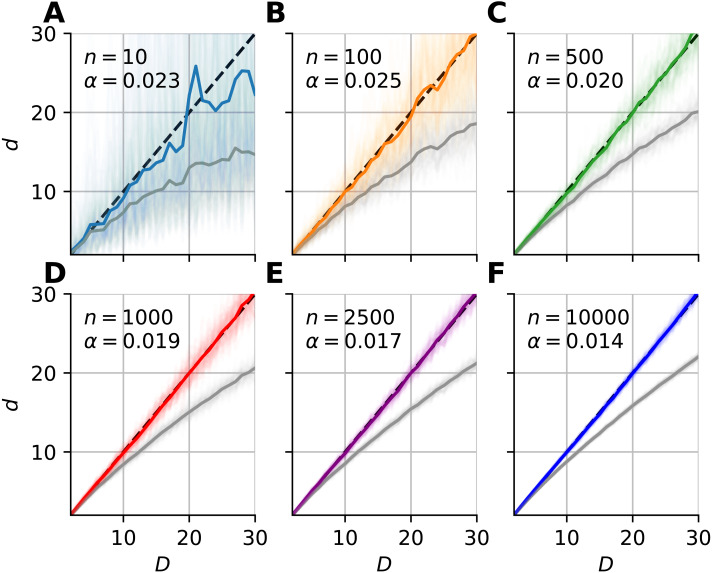
Bias-correction of the median-FSA estimator for uniformly sampled unit hypercubes with various sample sizes with hard boundary (*k* = 1). Subplots (A–F) show the mean of median-FSA estimator (grey line) values from *N* = 100 realizations (shading) of uniformly sampled unit hypercubes. The boundary condition is hard, so the edge effect makes under-estimation even more severe than in the case of periodic boundary condition. The colored lines show the corrected estimates according to the *d*_*c*_ = *d*exp(*αd*). In the *D* = 1–30 intrinsic dimension range a simple coefficient was enough to get small mean squared error after model fit.

We fitted a correction formula on the logarithm of dimension values and estimates with the least squares method (Eq. 10), using all 100 realizations for each sample sizes separately (Fig. 6).

(10) \begin{eqnarray*}\alpha = \frac{\sum (\ln \nolimits {E}_{i}){d}^{(i)}}{\sum { \left( {d}^{(i)} \right) }^{2}} \end{eqnarray*}

where *E*_*i*_ = *D*_*i*_/*d*^(*i*)^ is the relative error, *D*_*i*_ is the intrinsic dimension of the data, and *d*^(*i*)^ are the corresponding mFSA estimates. We carried out the model fit on the 2–30 intrinsic dimension range.

We also calibrated the cmFSA algorithm in a wider range of intrinsic dimension values (2–80) and applied more coefficients in the polynomial fit procedure (Fig. S1A). Also, we used orthogonal distance regression to fit the mean over realizations of ln*E*_*i*_ with the same *D*_*i*_ value (Fig. S1B). We utilized the mean and standard deviation of the regression error to compute the ideal error rate of cmFSA estimator, if the error-distributions are normal (Figs. S1C–S1F).

### Simulations on customly sampled manifolds

We carried out simulations on datasets sampled from manifolds according to uniform, multivariate Gaussian, Cauchy distribution and on uniformly sampled D-spheres in the function of sample size as in Facco et al. (2017), Fig. 2.

The uniform sampling was carried out on D-hypercube data with periodic boundary conditions. The Gaussian datasets were sampled from a zero mean and unit variance and no covariance multivariate normal distribution. The Cauchy datasets were generated so that the probability density of the norms were a Cauchy distribution. We achieved this by the following procedure:

1. Generate *n* points according to *D* dimensional Gaussian distribution (*ζ*_*i*_) and normalize the euclidean distance of the points from the origin.
\begin{eqnarray*}{z}_{i}= \frac{{\zeta }_{i}}{{|}{\zeta }_{i}{|}}  \text{where} {\zeta }_{i}\sim \mathcal{N}(0,I) \end{eqnarray*}
  and *I* is the *D*-dimensional identity matrix. Thus, the points *z*_*i*_ are uniformly distributed on the hyper-surface of a *D* − 1 dimensional hyper-sphere of unit radius.
2. Generate *n* positive real numbers *u*_*i*_ from a Cauchy distribution $f(u)= \frac{1}{\pi (1+{u}^{2})} $ and multiply *z*_*i*_ by this to get a dataset:
\begin{eqnarray*}{x}_{i}={u}_{i}\times {z}_{i} \end{eqnarray*}
  Thus the norms of the resulting points are distributed according to a Cauchy distribution.

Finally, we produced the D-sphere data with the first step of the previous procedure.

**Figure 7:**
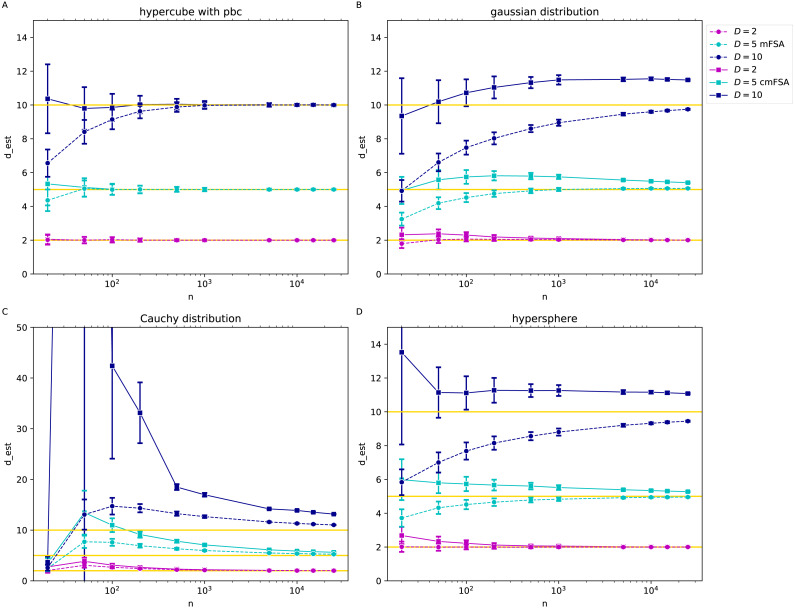
mFSA and cmFSA dimension estimates on customly sampled data in the function of sample size (*k* = 5, *D* = 2, 5, 10). The figure presents that mFSA and cmFSA makes errors if the sampling process is not uniform. (A) Results on hypercubes with periodic boundary conditions dataset shows, that mFSA systematically underestimates the intrinsic dimension especially for higher dimension values, this bias is corrigated by cmFSA. (B) The mFSA algorithm underestimates and cmFSA overestimates the intrinsic dimension for the Gaussian datasets. (C) For the Cauchy datasets, mFSA estimator shows an average underestimation at small sample sizes and an over-estimation region followed by convergence to true dimension value. cmFSA severely overestimates the intrinsic dimension values. (D) On the slightly curved hypersphere datasets mFSA also underestimates the intrinsic dimension and cmFSA gives and overestimation.

We generated *N* = 200 instances of each dataset with the intrinsic dimension values *D* = 2, 5, 10, we estimated the global mFSA and cmFSA dimensions and plotted the mean and standard deviation in the function of sample size (Fig. 7).

### Comparison on synthetic benchmark datasets

We simulated *N* = 100 instances of 15 manifolds (Table 1, *M*_*i*_, *n* = 2, 500) with various intrinsic dimensions. We generated the datasets according to the first 15 manifolds proposed by Campadelli et al. (2015). More specifically, Table 1 contains the description manifold types, the first 15 manifolds of Table 2 are used in this work as synthetic benchmark, and the Table 4 shows the benchmark results in Campadelli et al. (2015), http://www.mL.uni-saarland.de/code/IntDim/IntDim.htm.

**Table 1:** Synthetic benchmark datasets. The synthetic benchmark datasets used for comparison are the first 15 manifolds from Campadelli et al. (2015). The datasets represent various types of manifolds with or without curvature, also with uniform or non-uniform sampling of *n* = 2, 500 points.

	Dataset	Description	d	embed-dim
1	*M*_1_	10d sphere	10	11
2	*M*_2_	3d affine space	3	5
3	*M*_3_	4 figure	4	6
4	*M*_4_	4d manifold in 8d	4	8
5	*M*_5_	2d helix in 3d	2	3
6	*M*_6_	6dim manifold in 36d	6	36
7	*M*_7_	swiss roll	2	3
8	*M*_9_	20d affine space	20	20
9	*M*_10*a*_	10d hypercube	10	10
10	*M*_10*b*_	17d hypercube	17	17
11	*M*_10*c*_	24d hypercube	24	24
12	*M*_10*d*_	70d hypercube	70	70
13	*M*_11_	Moebius band 10x twisted	2	3
14	*M*_12_	Multivariate Gaussian	20	20
15	*M*_13_	1d curve in 13d	1	13

**Table 2:** Dimension estimates on synthetic benchmark datasets. The table shows true dimension values (d), median-Farahmand-Szepesvári-Audibert (mFSA), corrected median Farahmand-Szepesvári-Audibert (cmFSA), DANCo, Maximum Likelihood (Levina) and 2NN mean estimates from *N* = 100 realizations. cmFSA and DANCo was applied in integer and in fractal modes. The mean percentage error (MPE) values can be seen in the bottom line, the Matlab version of DANCo estimator (DANCo M) produced the smallest error followed by the cmFSA estimator.

Dataset	d	mFSA	cmFSA frac	cmFSA	DANCo R	DANCo M frac	DANCo M	Levina	2NN
*M*_1_	10	9.09	11.19	11.08	11.34	10.42	10.30	10.15	9.40
*M*_2_	3	2.87	3.02	3.00	3.00	2.90	3.00	3.20	2.93
*M*_3_	4	3.83	4.14	4.00	5.00	3.84	4.00	4.29	3.87
*M*_4_	4	3.95	4.29	4.00	5.00	3.92	4.00	4.38	3.91
*M*_5_	2	1.97	2.00	2.00	2.00	1.98	2.00	2.19	1.99
*M*_6_	6	6.38	7.38	7.16	9.00	6.72	7.00	7.04	5.93
*M*_7_	2	1.95	1.98	2.00	2.00	1.96	2.00	2.18	1.98
*M*_9_	20	14.58	20.07	20.10	19.16	19.24	19.09	16.38	15.55
*M*_10*a*_	10	8.21	9.90	10.00	10.00	9.56	9.78	9.20	8.63
*M*_10*b*_	17	12.76	16.95	16.96	16.04	16.39	16.24	14.33	13.58
*M*_10*c*_	24	16.80	24.10	24.06	23.61	23.39	23.26	18.89	18.04
*M*_10*d*_	70	35.64	69.84	69.84	69.73	71.00	70.91	40.35	40.05
*M*_11_	2	1.97	2.00	2.00	2.00	1.97	2.00	2.19	1.98
*M*_12_	20	15.64	21.96	21.98	21.72	20.88	20.00	17.72	17.24
*M*_13_	1	1.00	0.96	1.00	1.00	1.00	1.00	1.11	1.00
MPE		13.58	4.73	2.89	9.64	3.39	2.35	13.23	10.91

We applied the wide (*D* =2–80) calibration procedure (*l*_1_ =  − 1, *l*_2_ = 1, *l*_3_ = 2, *l*_4_ = 3) as in the previous subsection (*n* = 2, 500, *k* = 5) to compute cmFSA for the datasets. We used cmFSA in two modes, in integer and in fractal mode. In the former the global estimates are rounded to the nearest integer value, while in the latter case the estimates can take on real values.

We measured the performance of the mFSA and corrected-mFSA estimators on the benchmark datasets, and compared them with the performance of ML (Levina & Bickel, 2004) DANCo (Ceruti et al., 2014) and the 2NN (Facco et al., 2017) (Table 2) estimators. We used the Matlab (MATLAB, 2020; Lombardi, 2020) (see on github) and an R package (Johnsson, Soneson & Fontes, 2015) implementation of DANCo. In the case of DANCo, we also investigated the results for integer and for fractal mode just as for the cmFSA algorithm.

To quantify the performance we adopted the Mean Percentage Error (MPE, Eq. 11) metric (Campadelli et al., 2015):

(11) \begin{eqnarray*}\mathrm{MPE}= \frac{100}{MN} \sum _{j=1}^{M}\sum _{i=1}^{N} \frac{{|}{D}_{j}-{d}_{ij}{|}}{{D}_{j}} \end{eqnarray*}

where there is *N* realizations of *M* types of manifolds, *D*_*j*_ are the true dimension values, *d*_*ij*_ are the dimension estimates.

**Figure 8:**
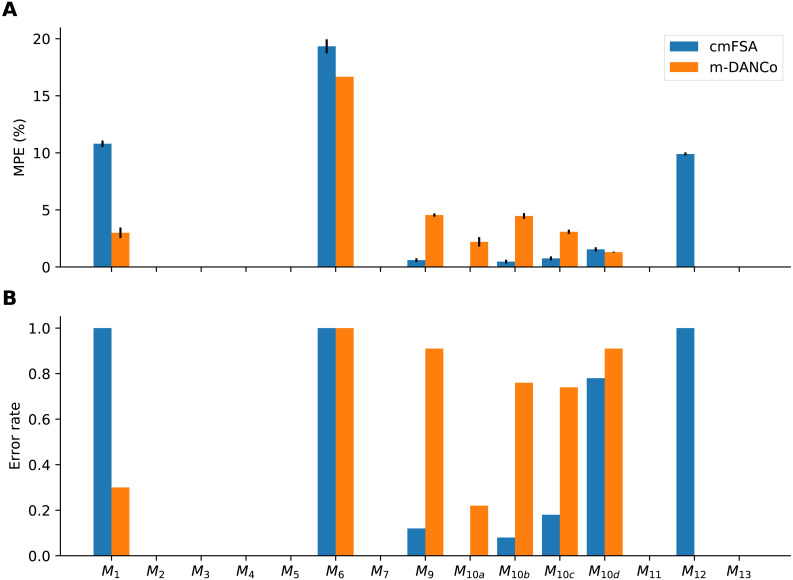
Performance-comparison between cmFSA and DANCo on synthetic benchmark datasets. cmFSA and DANCo have comparable performance with small differences according to Mean Percentage Error and Error rate metrics. (A) Dataset-wise Mean Percentage Error (MPE) on benchmark data. cm-FSA (blue) shows smaller MPE in 4 cases (*M*_9_, *M*_10*a*−*c*_) and bigger MPE in 4 cases (*M*_1_, *M*_6_, *M*_10*d*_, *M*_12_) compared with DANCo (Matlab). (B) Dataset-wise error rate for cmFSA and DANCo. cmFSA shows smaller error rates in 5 cases (*M*_9_, *M*_10*a*−*d*_) and bigger error rates in 2 cases (*M*_1_, *M*_12_) compared with DANCo.

Also, we used the error rate—the fraction of cases, when the estimator did not find (missed) the true dimensionality—as an alternative metric (Fig. 8). We used this metric to compare the performace of DANCo and cmFSA in integer mode, we simply counted the cases, when the estimator missed the true dimension value:

(12) \begin{eqnarray*}{H}_{j}= \frac{1}{N} \sum _{i=1}^{N}I({D}_{j}\not = {d}_{ij})\end{eqnarray*}

where *H*_*j*_ is the error rate for a manifold computed from *N* realizations and *I* = 1 if *D*_*j*_ ≠ *d*_*ij*_ is the indicator function for the error. We computed the mean error rate *H* by averaging the manifold specific values.

### Dimension estimation of interictal and epileptic dynamics

**Figure 9:**
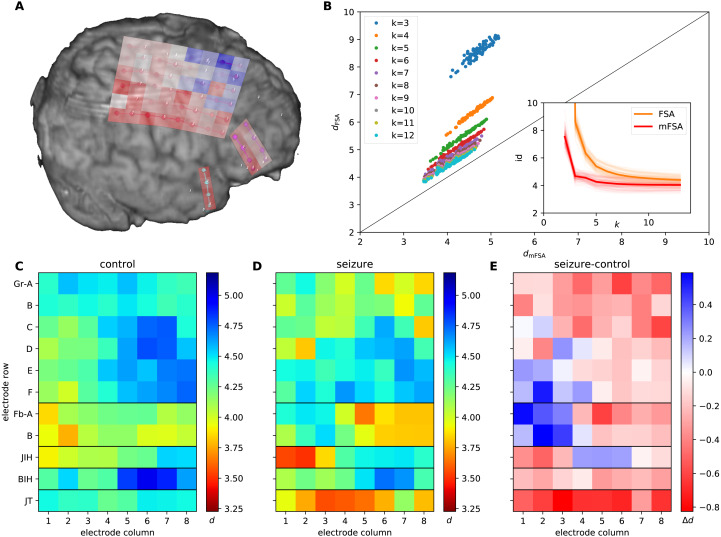
mFSA and FSA dimension estimates on intracranial brain-LFP measurements during interictal activity and epileptic seizures. (A) The experimental setup with the implanted electrodes are shown. A 64 channel intracranial cortical grid (red grid on graph A, Gr A1-F8 on graph C), a smaller frontobasal grid (magenta dots, Fb A1-B8) and a right temporal electrode strip, close to the hippocampus (cyan dots, JT1-8). Dimension estimates were calculated for two additional electrode strips close to the gyrus cinguli (JIH and BIH) which are hidden on this figure. The change in the mFSA estimates between seizure and control is color coded and mapped onto the recording electrodes. (B) Comparison of mFSA and FSA estimates on an epileptic seizure. FSA results in higher estimates, but the difference decreases with the increasing neighbourhood parameter *k*. (C) Average of mFSA dimension values from interictal LFP activity (*N* = 16, k =5–10). The areas with lower-dimensional dynamics are marked by hot colors. (D) Average of mFSA dimension values from seizure LFP activity (*N* = 18, k =5–10), colors same as on graph C. (E) Difference of average dimension values. Stronger red color marks areas, where the dynamics during seizure was smaller-dimensional than its interictal counterpart. However, stronger blue indicates electrodes, where the during-seizure dynamics was higher dimensional than the interictal dynamics.

We used data of intracranial field potentials from two subdural grids positioned –parietofrontally (6*8 channels, Gr A-F and 1–8) and frontobasally (2*8 channels, Fb A-B and 1–8) –on the brain surface and from three strips located on the right temporal cortex (8 channels, JT 1–8), close to the hippocampal formation and two interhemispheric strips, located within the fissura longitudinalis, close to the left and right gyrus cinguli (8 channels BIH 1–8 and 8 channels JIH 1–8) as part of presurgical protocol for a subject with drug resistant epilepsy (Fig. 9A). The participants signed a written consent form and the study was approved by the relevant institutional ethical committee (Medical Research Council, Scientific and Research-Ethics Committee TUKEB, Ref number: 20680-4/2012/EKU (368/PI/2012)). This equipment recorded extracellular field potentials at 88 neural channels at a sampling rate of 2048 Hz. Moreover, we read in—using the neo package (Garcia et al., 2014)—selected 10 second long chunks of the recordings from interictal periods (*N* = 16) and seizures (*N* = 18) to further analysis.

We standardised the data series and computed the Current Source Density (CSD) as the second spatial derivative of the recorded potential. We rescaled the 10 second long signal chunks by subtracting the mean and dividing by the standard deviation. Then, we computed the CSD of the signals by applying the graph Laplacian operator on the time-series. The Laplacian contains information about the topology of the electrode grids, to encode this topology, we used von Neumann neighborhood in the adjacency matrix. After CSD computation, we bandpass-filtered the CSD signals (Gramfort et al., 2013) (1–30 Hz, fourth order Butterworth filter) to improve signal to noise ratio.

We embedded CSD signals and subsampled the embedded time series. We used an iterative manual procedure to optimize embedding parameters (Fig. S2). Since the fastest oscillation is (30 Hz) in the signals, a fixed value with one fourth period (2048/120 ≈ 17 samples) were used as embedding delay. We inspected the average space–time separation plots of CSD signals to determine a proper subsampling, with the embedding dimension of *D* = 2 (Fig. S2A). We found, that the first local maximum of the space–time separation was at around 5 ms: 9–10, 10–11, 11–12 samples for the 1%, 25%, 50% percentile contour-curves respectively. Therefore, we divided the embedded time series into 10 subsets to ensure the required subsampling. Then, we embedded the CSD signal up to *D* = 12 and measured the intrinsic dimensionality for each embeddings (Figs. S2B and S2C). We found that intrinsic dimension estimates started to show saturation at *D* ≥ 3, therefore we chose *D* = 7 as a sufficiently high embedding dimension (averaged over *k* =10–20 neighborhood sizes).

We measured the intrinsic dimensionality of the embedded CSD signals using the mFSA method during interictal and epileptic episodes (Fig. 9). We selected the neighborhood size between *k* = 10 and *k* = 20 and averaged the resulting estimates over the neighborhoods and subsampling realizations. We investigated the dimension values (Figs. 9C and 9D) and differences (Fig. 9E) between interictal and epileptic periods.

We also compared the mFSA estimates with the original –mean based –FSA estimates in the function of neighborhood size on a recording in the *k* =1–12 neighborhood range and plotted the estimates against each other to visualize differences (Fig. 9B).

## Results

### Manifold adaptive dimension estimator revisited

#### The probability density of Farahmand-Szepesvári-Audibert estimator

We compute the probability density function of Farahmand-Szepesvári-Audibert (FSA) intrinsic dimension estimator based on normalized distances.

The normalized distance density of the *k*NN can be computed in the context of a *K*-neighborhood, where the normalized distance of *K* − 1 points follows a specific form:

(13) \begin{eqnarray*}p(r{|}k,K-1,D)= \frac{D}{B(k,K-k)} {r}^{Dk-1}(1-{r}^{D})^{K-k-1}\end{eqnarray*}

where *r* ∈ [0, 1] is the normalized distance of the *k*th neighbor and *B* is the Euler-beta function. In practice, the normalization is carried out by dividing with the distance of *K*th neighbor (*r*_*k*_ = *R*_*k*_/*R*_*K*_, *k* < *K*). Here *p*(*r*|*k*, *K* − 1, *D*)Δ*r* describes the probability that the *k*-th neighbor can be found on a thin shell at the normalized distance *r* among the *K* − 1 neighbors if the intrinsic dimension is *D* (see SI A. 1 for a derivation). A maximum likelihood estimator based on Eq. (13) leads to the formula of the classical Levina-Bickel estimator (Levina & Bickel, 2004). For a derivation of this probability density and the maximum likelihood solution see SI A. 1 and SI A. 2 respectively.

We realize that the inverse of normalized distance appears in the formula of FSA estimator, so we can express it as a function of *r*:

(14) \begin{eqnarray*}{\delta }_{k}= \frac{\log \nolimits 2}{\log \nolimits \left( {R}_{2k}/{R}_{k} \right) } =- \frac{\log \nolimits 2}{\log \nolimits \left( {R}_{k}/{R}_{2k} \right) } =- \frac{\log \nolimits 2}{\log \nolimits {r}_{k}} \end{eqnarray*}

Where *r*_*k*_ = *R*_*k*_/*R*_2*k*_.

Combining Eqs. (13) and (14), one can obtain the pdf of the FSA estimator:

(15) \begin{eqnarray*}q \left( {\delta }_{k} \right) \equiv p \left( r{|}k,2k-1,D \right) \left\vert \frac{dr}{d{\delta }_{k}} \right\vert = \frac{D\log \nolimits (2)}{B(k,k)} \frac{{2}^{- \frac{Dk}{{\delta }_{k}} }{ \left( 1-{2}^{- \frac{D}{{\delta }_{k}} } \right) }^{k-1}}{{\delta }_{k}^{2}} \end{eqnarray*}

Theorem 1 *The median of q*(*δ*_*k*_)*is at D*.

Proof We apply the monotonic substitution *a* = 2^−*D*/*δ*_*k*_^ on Eq. (15):

(16) \begin{eqnarray*}p(a)& =q({\delta }_{k}) \left\vert \frac{d{\delta }_{k}}{da} \right\vert =\nonumber\\\displaystyle & \end{eqnarray*}

(17) \begin{eqnarray*}= \frac{D\log \nolimits (2)}{B(k,k)} \frac{{a}^{k}(1-a)^{k-1}{\log \nolimits }^{2}a}{{D}^{2}{\log \nolimits }^{2}2} \frac{D\log \nolimits 2}{a{\log \nolimits }^{2}a} \nonumber\\\displaystyle & \end{eqnarray*}

(18) \begin{eqnarray*}= \frac{1}{B(k,k)} {a}^{k-1}(1-a)^{k-1}\end{eqnarray*}

The pdf in Eq. (18) belongs to a beta distribution. The cumulative distribution function of this density is the regularized incomplete Beta function (*I*_*a*_) with *k* as both parameters symmetrically.

(19) \begin{eqnarray*}P(a)={I}_{a}(k,k)\end{eqnarray*}

The median of this distribution is at $a= \frac{1}{2} $, thus at *δ*_*k*_ = *D*since:

(20) \begin{eqnarray*}a={2}^{- \frac{D}{{\delta }_{k}} }& =& \frac{1}{2} \end{eqnarray*}

(21) \begin{eqnarray*}D& =& {\delta }_{k}\end{eqnarray*}

and *a* is a monotonic function of *δ*, therefore the median in *δ*_*k*_ can be computed by the inverse mapping. □

This means that the median of the local FSA estimator is equal to the intrinsic dimension independent of neighborhood size, even for the minimal neighborhood, if the locally uniform point density assumption holds. The sample median is a robust statistic, therefore we propose to use the sample median of local estimates as a global dimension estimate. We will call this modified method the ’median Farahmand-Szepesvári-Audibert’ (mFSA) estimator.

Let’s see the form for the smallest possible neighborhood size: *k* = 1 (Fig. 2). The pdf for the estimator takes a simpler from Eq. (22).

(22) \begin{eqnarray*}q(\delta {|}k=1,D)=D\log \nolimits (2) \frac{{2}^{- \frac{D}{{\delta }_{1}} }}{{\delta }_{1}^{2}} \end{eqnarray*}

Also, we can calculate the cumulative distribution function analytically (Eq. 23).

(23) \begin{eqnarray*}Q(\delta {|}k=1,D)=\int \nolimits \nolimits _{0}^{{\delta }_{1}}q(t{|}k=1,D) dt={2}^{-D/{\delta }_{1}}\end{eqnarray*}

The expectation of *δ*_*k*_ diverges for *k* = 1–but not for *k* > 1 –although the median exists.

(24) \begin{eqnarray*}Q({\delta }_{1}=D)=\int \nolimits \nolimits _{0}^{D}q(t{|}k=1,D) dt=0.5\end{eqnarray*}

From Eq. (23) the median is at *D* Eq. (24).

#### Sampling distribution of the median

We can compute the pdf of the sample median if an odd sample size is given (*n* = 2*l* + 1) and if sample points are drawn independently according to Eq. (15) (see Supplemental Information 1 secC for a derivation). Roughly half of the points have to be smaller, half of the points have to be bigger and one point has to be exactly at *d* (Eq. 25).

(25) \begin{eqnarray*}p(d{|}k,D,n)= \frac{1}{B(l+1,l+1)} { \left[ P \left( a={2}^{-D/d} \right) \left( 1-P \left( a={2}^{-D/d} \right) \right) \right] }^{l}q(d)\end{eqnarray*}

where *p*(*a*) and *P*(*a*) are the pdf and cdf of *a* (Eqs. (18), (19)) and *q* is the pdf of the FSA estimator (Figs. 3A, 3B).

We determine the standard error by the numerical integration of Eq. (25) and found that the error shrinks approximately with the square-root of *n* and *k* (Figs. 3C, 3D). Also, the value of the standard error is proportional to the dimension of the manifold. From these observations, we express the error as:

(26) \begin{eqnarray*}{\sigma }_{d}\approx \kappa \frac{D}{\sqrt{nk}} \end{eqnarray*}

where *κ* is a constant. These empirical results can be backed up by theory: the same expression arises for the standard error by using the Laplace and Stirling approximations, also by these methods, the exact value of $\kappa = \frac{\sqrt{\pi }}{2\log 2} $ can be derived (see Supplemental Information 1 secD for a derivation).

#### Maximum Likelihood solution for the manifold-adaptive estimator

If the samples are independent and identically distributed, we can formulate the likelihood function as the product of sample-likelihoods (Eq. (27)). We seek for the maximum of the log likelihood function, but the derivative is transcendent for *k* > 1. Therefore, we can compute the place of the maximum numerically (Eq. (29)).

(27) \begin{eqnarray*}\mathcal{L}& =& \prod _{i=1}^{n} \frac{D\log \nolimits (2)}{B(k,k)} \frac{{2}^{-Dk/{\delta }^{(i)}}(1-{2}^{-D/{{\delta }_{k}}^{(i)}})^{k-1}}{{ \left( {{\delta }_{k}}^{(i)} \right) }^{2}} \end{eqnarray*}

(28) \begin{eqnarray*}\log \nolimits \mathcal{L}& =& n\log \nolimits \frac{\log \nolimits (2)}{B(k,k)} +n\log \nolimits D-Dk\log \nolimits (2)\sum \frac{1}{{{\delta }_{k}}^{(i)}} +(k-1)\sum \log \nolimits \left( 1-{2}^{-D/{{\delta }_{k}}^{(i)}} \right) \nonumber\\\displaystyle & & -2\sum \log \nolimits ({{\delta }_{k}}^{(i)})\end{eqnarray*}

(29) \begin{eqnarray*} \frac{\partial \log \nolimits \mathcal{L}}{\partial D} & =& \frac{n}{D} -\log \nolimits (2)k\sum \frac{1}{{{\delta }_{k}}^{(i)}} +\log \nolimits (2)(k-1)\sum \frac{1}{{{\delta }_{k}}^{(i)}({2}^{D/{{\delta }_{k}}^{(i)}}-1)} \stackrel{{!}}{=}0\end{eqnarray*}

For *k* = 1, the ML formula is equal to the Levina-Bickel (*k* = 1) and MIND_1ML_ formulas.

### Results on randomly sampled hypercube datasets

Theoretical probability density function of the local FSA estimator fits to empirical observations (Eq. (15), Fig. 2). We simulated hypercube datasets with fixed sample size (*n* = 10, 000) and of various intrinsic dimensions (*D* = 2, 3, 5, 8, 10, 12). We measured the local FSA estimator at each sample point with three different *k* parameter values (*k* = 1, 11, 50). We visually confirmed that the theoretical pdf fits perfectly to the empirical histograms.

The empirical sampling distribution of mFSA fits to the theoretical curves for small intrinsic dimension values (Fig. 3). To demonstrate the fit, we drew the density of mFSA on two hypercube datasets *D* = 2 and *D* = 5 with the smallest possible neighborhood (*k* = 1), for different sample sizes (*n* = 11, 101, 1,001). At big sample sizes the pdf is approximately a Gaussian (Laplace, 1986), but for small samples the pdf is non-Gaussian and skewed.

The mFSA estimator underestimates intrinsic dimensionality in high dimensions. This phenomena is partially a finite sample effect (Fig. 4), but edge effects make this underestimation even more severe. This phenomenon was pronounced at low sample sizes and high dimensions, but we experienced convergence to the real dimension value as we increased sample size.

We graphically showed that mFSA estimator asymptotically converged to the real dimension values for hypercube-datasets, when we applied periodic boundary conditions (Fig. 5). We found, that the convergence is much slower for hard boundary conditions, where edge effects make systematic estimation errors higher.

From the shape of the curves in Fig. 4, we heuristically derived a correction formula for finite sample size and edge effects (Eq. 9). The heuristics is as follows. We tried to find a formula, which intuitively describes the true intrinsic dimension in the function (*C*) of the estimated values. One can see on Fig. 4, that at small values the error converges to zero and also the curve lies approximately on the diagonal, so it’s derivative goes to one.

(30) \begin{eqnarray*}\lim _{d\rightarrow 0}C(d) & =D\lim _{d\rightarrow 0}{C}^{{^{\prime}}}(d) & =1\end{eqnarray*}

where *C* is the correction function and *d* is the biased estimate. Equation (9) satisfies these conditions and gives good fit to empirical data (Fig. 6).

From an other point of view, Eq. (9) means that one could estimate the logarithm of relative error with an *L*-order polynomial:

(31) \begin{eqnarray*}\log \nolimits ({E}_{rel})=\log \nolimits \left( \frac{D}{d} \right) =\sum _{l=1}^{L}{\alpha }_{l}{d}^{l}\end{eqnarray*}

The order of the polynomial was different for the two types of boundary conditions. When we applied hard boundary, the order was *L* = 1, however in the periodic case higher order polynomials fit the data. Thus, in the case of hard-boundary, we could make the empirical correction formula:

(32) \begin{eqnarray*}D\approx C(d)=d{e}^{{\alpha }_{n}d}\end{eqnarray*}

where *α*_*n*_ is a sample size dependent coefficient that we could fit with the least squares method. This simple model described well the data in the 2–30 intrinsic dimension range (Figs. 6A–6F).

### Results on customly sampled manifolds

We investigated the case when the assumption of uniform sampling or flatness is violated through gaussian, Cauchy and hypersphere datasets (Fig. 7) with various intrinsic dimensions and sample sizes. We added hypercube datasets with periodic boundary conditions as a control with the same parameter setting respectively (*k* = 5).

On the hypercube datasets with periodic boundary conditions the mFSA algorithm produced a massive underestimation of intrinsic dimension for low sample sizes for *D* = 10, but cmFSA corrects this bias caused by finite sample size (Fig. 7A). For the small-dimensional cases. When *D* = 2 and *D* = 5 both cmFSA and FSA estimated well the true intrinsic dimension values. On the gaussian datasets with non-periodic boundary conditions mFSA produced even more severe underestimation for *D* = 10 or *D* = 5, but cmFSA overestimated the intrinsic dimensions (Fig. 7B). On the heavy tailed Cauchy datasets mFSA showed a non-monotonic behaviour in the function of sample size: for fewer points it had low values with a maximum at mid sample sizes and exhibited slow decline convergence to true dimension value for big samples (Fig. 7C). This shape of the curve resulted in underestimation for small samples followed by an overestimation part depressing towards the true dimension values as *N* goes to infinity (*D* = 5, 10). For *D* = 2 the first underestimation section was missing. cmFSA produced severe overestimation for these Cauchy datasets. The hypersphere dataset is an example when the point density is approximately uniform, but the manifold is curved (Fig. 7D). On this datset mFSA produced underestimation for *D* = 5, 10 and good estimates for *D* = 2. cmFSA overestimated the dimension value.

### Results on synthetic benchmarks

We tested the mFSA estimator and its corrected version on synthetic benchmark datasets (Hein & Audibert, 2005; Campadelli et al., 2015). We simulated *N* = 100 instances of 15 manifolds (Table 1, *M*_*i*_, *n* = 2, 500) with various intrinsic dimensions.

We estimated the intrinsic dimensionality of each sample and computed the mean, the error rate and Mean Percentage Error (MPE) for the estimators. We compared the mFSA, cmFSA, the R and the Matlab implementation of DANCo, the Levina-Bickel and the 2NN estimator (Table 2). cmFSA and DANCo was evaluated in two modes, in a fractal-dimension mode and in an integer dimension mode.

The mFSA estimator underestimated intrinsic dimensionality, especially in the cases when the data had high dimensionality. The Levina-Bickel estimator overestimated low intrinsic dimensions and underestimated the high ones. The 2NN estimator produced underestimation on most test manifolds it reached the best average result on the *M*_6_ and *M*_13_ manifolds.

In contrast, the cmFSA estimator found the true intrinsic dimensionality of the datasets, it reached the best overall error rate (0.277) and 2nd best MPE (Fig. 8, Table 2). In some cases, it slightly over-estimated the dimension of test datasets. Interestingly, DANCo showed implementation-dependent performance, the Matlab algorithm showed the 2nd best error rate (0.323) and the best MPE value (Table 2). The R version overestimated the dimensionality of datasets in most cases.

### Analysing epileptic seizures

To show how mFSA works on real-world noisy data, we applied it to human neural recordings of epileptic seizures.

We acquired field potential measurements from a patient with drug-resistant epilepsy by 2 electrode grids and 3 electrode strips. We analyzed the neural recordings during interictal periods and during epileptic activity to map possible seizure onset zones (see Methods).

We found several characteristic differences in the dimension patterns between normal and control conditions. In interictal periods (Fig. 9C), we found the lowest average dimension value at the FbB2 position on the fronto-basal grid. Also, we observed gradually increasing intrinsic dimensions on the cortical grid (Gr) between the F1 and D6 channels. In contrast, we observed the lowest dimension values at the right interhemispherial strip (JIH 1–2) and on the temporo-basal electrode strip (JT 3–5) close to the hippocampus, and the gradient on the cortical grid altered during seizures (Fig. 9D). Comparing the dimensions between seizure and control periods, the majority of the channels showed lower dimensions during seizures. This decrease was most pronounced close to the hippocampal region (strip JT) and the parietal region mapped by the main electrode grid (GrA-C). Curiously, the intrinsic dimensionality became higher at some frontal (GrE1-F2) and fronto-basal (FbA1-B3) recording sites during seizure (Figs. 9A and 9E).

Comparison of the original FSA and the mFSA dimension estimators on the seizure data series showed characteristic difference similar to the one observed in the simulated data: mFSA resulted in lower dimension estimates than FSA and the difference between the two methods decreases as the *k* neighbourhood increases (Fig. 9B, compare it with Figs. 1C and 1D).

## Discussion

In this work we revisited and improved the manifold adaptive FSA dimension estimator. We computed the probability density function of local estimates for uniform local density. From the pdf, we derived the maximum likelihood formula for intrinsic dimensionality. However these results were derived for the simplest uniform euclidean manifold with single global intrinsic dimension, they form a base for application in more complex cases. For example the pdf of the local statistic make possible to apply the FSA estimator within mixture-based approaches, this would provide better ID estimates when the ID is varying in the data set (Haro, Randall & Sapiro, 2008; Allegra et al., 2020).

We proposed to use the median of local estimates as a global measure of intrinsic dimensionality, and demonstrated that this measure is asymptotically unbiased. This property holds even for the minimal *k* = 1 neighborhood size, where the previously proposed mean is infinite. The use of minimal neighborhood may be relevant, because it ameliorates the effect of curvature and density inequalities (Facco et al., 2017).

We tackled edge and finite sample effects with a correction formula calibrated on hypercube datasets. We showed that the coefficients are sample-size dependent. Camastra and Vinciarelli (Camastra & Vinciarelli, 2002) took a resembling empirical approach, where they corrected correlation dimension estimates with a perceptron, calibrated on d-dimensional datasets. Our approach is different, because we tried to grasp the connection between underestimation and intrinsic dimensionality more directly, by showing that the dimension-dependence of the relative error is exponential (Eq. 31). The calibration procedure of DANCo may generalize better, because it compares the full distribution of local estimates rather than just a centrality measure (Ceruti et al., 2014). Also, we are aware that our simple correction formula overlooks the effect of curvature, uneven density and noise. One can try to address the effect of curvature and nonuniform density with the choice of minimal neighborhood size (*k* = 1), thus the estimation error is minimal (Facco et al., 2017). We investigated cases when the flatness and uniformity assumptions is violated on curved and unevenly sampled manifolds as in Facco et al. (2017) and found that the estimation errors can be large both for mFSA and cmFSA. We investigated the non-uniform sampling with Gaussian and Cauchy datasets (*k* = 5). For the Gaussian dataset cmFSA moderately overestimated the values. For the Cauchy dataset the overestimation of cmFSA is very severe: for less than 500 points, the estimation error and also the standard deviation seems to be unbounded. On the curved hypersphere data cmFSA also produced moderate overestimation. These datasets are quite challenging, and the 2NN method of Facco et al. (2017), which uses minimal neighborhood information, presents more exact results on these. The simplicity of the correction in cmFSA, more specifically that the calibration is based on uniformly sampled hypercube datasets makes it vulnerable to non-uniform density and curvature. Additionally, the effect of noise on the estimates is yet to be investigated. There are several strategies to alleviate noise effects such as undersample the data while keeping the neighborhood fixed (Facco et al., 2017), or using a larger neighborhood size, while keeping the sample size fixed. Both of these procedures make the effect of curvature more severe, which makes the dimension estimation of noisy curved data a challenging task.

We benchmarked the new mFSA and corrected-mFSA method against Levina-Bickel estimator, 2NN and DANCo on synthetic benchmark datasets and found that cmFSA showed comparable performance to DANCo. For many datasets, R-DANCo overestimated the intrinsic dimensionality, which is most probably due to rough default calibration (Johnsson, Soneson & Fontes, 2015); the Matlab implementation showed the best overall results in agreement with Campadelli et al. (2015). This superiority was however dataset-specific: cmFSA performed genuinely the best in 2, DANCo in 1 out of the 15 benchmark datasets, with 7 ties (Table 2). Also, cmFSA showed better overall error rate than DANCo. Combining the performance measured by different metrics, we recognise that cmFSA found the true intrinsic dimension of the data in more cases, but when mistaken, it makes relatively bigger errors compared with DANCo. More specifically in the cases of *M*_1_, *M*_6_, *M*_12_ cmFSA almost never hits the true intrinsic dimension value, where *M*_1_ is a 10-dimensional sphere, *M*_6_ is a 6-dimensional manifold embedded in 36 dimensions and *M*_12_ is a 20-dimensional multivariate Gaussian. In the first case the manifold is curved, in the second it is embedded in high dimensional ambient space and in the third one it is non-uniformly sampled. DANCo was robust against the curvature and the non-uniform sampling, but also exhibited vulnerability to high ambient space data *M*_6_. For this dataset the 2NN method performed the best.

The mFSA algorithm revealed diverse changes in the neural dynamics during epileptic seizures. In normal condition, the gradient of dimension values on the cortical grid reflects the hierarchical organization of neocortical information processing (Tajima et al., 2015). During seizures, this pattern becomes disrupted pointing to the breakdown of normal activation routes. Some channels showed lower dimensional dynamics during seizures; that behaviour is far from the exception: the decrease in dimensionality is due to widespread synchronization events between neural populations (Mormann et al., 2000), a phenomenon reported by various authors (Polychronaki et al., 2010; Bullmore et al., 1994; Päivinen et al., 2005).

Benkő et al. (2018) showed, that dimensional relations between time series from dynamical systems can be exploited to infer causal relations between brain areas. In the special case of unidirectional coupling between two systems, the dimension of the cause should be lower than the dimension of the consequence. Thus, the lower-dimensional areas are possible causal sources (Sugiyama & Borgwardt, 2013; Krakovská, 2019; Benkő et al., 2018) and candidates for being the seizure onset zone. Interestingly, Esteller et al. found, that the Higuchi fractal dimension values were higher at seizure onset and decreased to lower values as the seizures evolved over time (Esteller et al., 1999). We found, that most areas showed decreased dimensionality, but few areas also showed increased dimension values as seizure takes place. This may suggests that new - so far unused - neural circuits are activated at seizure onset; whether this circuitry contributes to or counteracts epileptic seizure is unclear.

## Conclusion

In this work we revisited the manifold adaptive dimension estimation problem, made improvements on the Farahmand-Szepevári-Audibert (FSA) intrinsic dimension estimator and applied the new algorithm on simulated and real-world datasets.

We derived the probability density function of local dimension estimates for uniform data density and proved that the median is an unbiased estimator of the global intrinsic dimension, even at small neighborhoods. Therefore, we proposed the use of median as a global dimension estimate as the median-FSA (mFSA) algorithm. We also wrote the expression to be optimized for the maximum likelihood solution.

We created a heuristic correction formula to tackle the bias caused by finite sample and edge effects. The resulting method is the corrected mFSA (cmFSA) algorithm, which corrigates the bias in the estimates according to an exponential formula calibrated on uniform hypercube datasets.

We compared the performance of the mFSA and cmFSA algorithms with the Levina-Bickel, 2NN and the DANCo estimators on benchmark datasets. We found that cmFSA showed comparable performance to DANCo.

We applied the mFSA algorithm to investigate the dynamics of human brain activity during epileptic seizures and resting state. We hypothesized that areas exhibiting low dimensional dynamics have important role as initiators or maintainers of seizure activity.

## Supplemental Information

10.7717/peerj-cs.790/supp-1 Supplemental Information 1 Supplemental derivations and figures to support some claims in the main texts

10.7717/peerj-cs.790/supp-2 Supplemental Information 2 Calibration procedure of cmFSA estimator for the *n* = 2, 500 datasets up to *D* = 80 (*k* = 5) The figure shows the calibration procedure on 100 instances of uniformly sampled hypercubes. A Dimension estimates in the function of intrinsic dimensionality for the calibration hypercubes. The diagonal (dashed) is the ideal value, however the mFSA estimates (blue) show saturation because of finite sample and edge effects. cmFSA estimates (red) are also shown, with the mean (yellow) almost aligned with the diagonal. B The relative error (E) in the function of uncorrected mFSA dimension on semilogarithmic scale. The error-mFSA pairs (blue) lie on a short stripe for each intrinsic dimension value. The subplot also shows id-wise average points (yellow) and the polynomial fitting curve (red). C The error of cmFSA estimates in the function of intrinsic dimension on the calibration datasets. The mean error (blue line) oscillates around zero and the 99.7 confidence interval (blue dashed) widens as ID grows. The rounding switch-points are also shown. D The probability that cmFSA hits the real ID of data, or misses by one, two or more as a function of ID on the calibration dataset. E The error is approximately gaussian as shown through the empirical distribution at *D* = 18 with the fitted gaussian. F Results of normality test show, that the error do not deviate significantly (alpha =0.05 dashed line) from a gaussian error distribution. We applied Bonferroni correction for multiple comparisons, the blue bars are the *p*-values.

10.7717/peerj-cs.790/supp-3 Supplemental Information 3 Subsampling and embedding of the CSD signals A Mean Space–time separation plot of the CSD recordings, the lines show the contours of the 1% (blue), 25% (orange), and 50% (green) percentiles for the 34–16 interictal and 18 seizures - recordings (thin lines) and their average (thick line, *D* = 2). The first local maximum is at around 5 ms (10 time steps), which appoints the proper subsampling to avoid the effect of temporal correlations during the dimension estimation. B Intrinsic dimension in the function of the embedding dimension for the 88 recording-channels (averaged between k =5–10, for the first seizure). Dimension-estimates deviate from the diagonal above *D* = 3, thus we chose D =2*3+1 =7 as embedding dimension. C Intrinsic dimension in the function of neighborhood size for various embedding dimensions (88 channels, for the first seizure). The dimension estimates are settled at the neighborhood size between k =10–20 (dashed blue). The knee because of the autocorrelation becomes pronounced for D≥8.

## Additional Information and Declarations

### Data Availability

The following information was supplied regarding data availability: All the code and raw data (including raw EEG data) is available at GitHub: https://github.com/phrenico/cmfsapy.
