# Supplementary material for: Manifold-adaptive dimension estimation revisited
Source: PeerJ Comput Sci. 2022 Jan 6;8:e790. doi: 10.7717/peerj-cs.790 (PMC8771813; doi:10.7717/peerj-cs.790)
Supplement: Supplemental Information 1 [file peerj-cs-08-790-s001.pdf]

Supplemental Information: Manifold-adaptive dimension estimation revisited

Zsigmond Benkő[1,2,*], Marcell Stippinger[1], Roberta Rehus[1], Attila Bencze[1], Dániel Fabó[4], Boglárka Hajnal[2,4], Loránd Erőss[5,6], András Telcs[1,3,7], and Zoltán Somogyvári[1,8]

[1]Department of Computational Sciences, Wigner Research Centre for Physics, H-1121, Hungary
[2]János Szentágothai Doctoral School of Neurosciences, Semmelweis University, H-1085, Hungary
[3]Department of Computer Science and Information Theory, Faculty of Electrical Engineering and Informatics, Budapest University of Technology and Economics, Budapest, H-1111, Hungary
[4]Epilepsy Center, Department of Neurology, National Institute of Clinical Neurosciences, Budapest, H-1145, Hungary
[5]Department of Functional Neurosurgery, National Institute of Clinical Neurosciences, Budapest, H-1145, Hungary
[6]Faculty of Information Technology and Bionics, Péter Pázmány Catholic University, Budapest, H-1083, Hungary
[7]Department of Quantitative Methods, University of Pannonia, Faculty of Business and Economics, H-8200, Veszprém, Hungary
[8]Neuromicrosystems ltd., Budapest, H-1113, Hungary

A Calculations for normalized distances

A.1 Distance density of the nearest neighbors

Let's take $K-1$ points in the unit D-sphere randomly, and dimensionality for the calibration hypercubes. The diagonal (dashed) is thewe chose one with r distance from the center. This situation simulates a K-neighborhood, with normalized distances of $K-1$ points from the center. The next formula tells us the probability that a selected point at r was the kth from the center.

$$P(k|r,K,D) = \binom{K-2}{k-1} r^{D(k-1)}(1-r^D)^{K-k-1} \tag{S.1}$$

here r can take values from the $[0,1]$ interval.

Moreover the probability density that there is a point at r radius is given by the following derivation formula:

$$p(r|D) = Dr^{D-1} \tag{S.2}$$

If sampling process is independent, the pdf that a point is on the radius r from $K-1$ points is the same and independent of sample size:

$$\begin{aligned} p(r|K-1,D) &= \sum_{j=1}^{n} \frac{1}{n} \underbrace{\int \mathrm{d}r_1 \cdots \int \mathrm{d}r_i \cdots \int \mathrm{d}r_n}_{i \neq j} p(r_1, r_2, \dots r_j = r, \dots r_n|D) \\ &= \sum_{j=1}^{n} \frac{1}{n} \underbrace{\int \int \cdots \int}_{n-1} \prod_{i=1}^{n} D{r_i}^{D-1} \underbrace{\mathrm{d}r_i}_{i \neq j} = \frac{1}{n} \sum_{j=1}^{n} D{r_j}^{D-1} = Dr^{D-1} \end{aligned} \tag{S.3}$$

This is the prior pdf of distance, we assume uniform density in the n-sphere. This prior can be any density, we chose this specific form with respect to the maximum entropy principle and also for practical reasons.

From the previous two formulas, we can write up the joint mixed probability function:

$$p(k, r|K-1, D) = D\binom{K-2}{k-1} r^{Dk-1}(1-r^D)^{n-k} \tag{S.4}$$

Also:

$$p(k|K-1, D) = \frac{1}{K-1} \tag{S.5}$$

Using Bayes theorem, we derive the distance distribution of the kth neighbor:

$$\begin{aligned} p(r|k, K-1, D) &= \frac{P(k|r, K-1, D)p(r|K-1, D)}{p(k|K-1, D)} && \text{(S.6)} \\ &= (K-1)D\binom{K-2}{k-1} r^{Dk-1}(1-r^D)^{K-k-1} && \text{(S.7)} \\ &= \frac{D}{B(k, K-k)} r^{Dk-1}(1-r^D)^{K-k-1} && \text{(S.8)} \end{aligned}$$

Where B is the beta function.

A.2 Maximum Likelihood estimation of intrinsic dimension

Given a dataset, we can use the Maximum Likelihood principle to estimate intrinsic dimensionality by using Eq. S.8. The dataset is $K-1$ randomly sampled points inside a d-dimensional sphere. But first we have to express the likelihood function:

$$\mathcal{L}(D|X) = p(r_1, ..., r_{K-1}|D) \tag{S.9}$$

This expression can be factorized into a chain because $p(r_k|r_{k+1}, r_{k+2}, ..., r_{K-1}) = p(r_k|r_{k+1})$ which is a Markov property of neighbor distances.

$$\mathcal{L}(D|X) = p(r_1, ..., r_{K-1}|D) = \prod_1^{K-1} p(r_k|r_{k+1}, D) \tag{S.10}$$

where $r_K = 1$.

$$p(r_k|r_{k+1}, D) = kD \left(\frac{r_k}{r_{k+1}}\right)^{kD-1} \frac{1}{r_{k+1}} \tag{S.11}$$

So if we substitute back into the previous expression:

$$\begin{aligned} \mathcal{L}(D|X) = p(r_1, ..., r_n|D) &= \prod_1^{K-1} p(r_k|r_{k+1}, d) \\ &= (K-1)!\, D^{K-1} \frac{r_1^{D-1}}{r_2^D} \frac{r_2^{2D-1}}{r_3^{2D}} \frac{r_3^{3D-1}}{r_4^{3D}} \cdots \frac{r_{K-1}^{(K-1)D-1}}{r_K^{(K-1)D}} \\ &= (K-1)!\, D^{K-1} \left(\prod_1^{K-1} r_k\right)^{D-1} \end{aligned} \tag{S.12}$$

The log likelihood:

$$\log \mathcal{L}(D|X) = \left(\sum_1^{K-1} \log k\right) + (K-1)\log D + (D-1)\sum_1^{K-1} \log r_k \tag{S.13}$$

We seek for extrema of the likelihood function:

$$\begin{aligned} \frac{\partial \log \mathcal{L}(D|X)}{\partial D} &\overset{!}{=} 0 \\ \frac{K-1}{D} + \sum_1^{K-1} \log r_k &\overset{!}{=} 0 \end{aligned} \tag{S.14}$$

$$\boxed{d_{\mathrm{ML}} = \frac{K-1}{-\sum_1^{K-1} \log r_k}} \tag{S.15}$$

This latter formula is basically equivalent to the local Levina-Bickel ML intrinsic dimension estimator if $r_k = \frac{R_k}{R_K}$.

B Derivation of the pdf of the FSA estimator

The starting point of our derivation is the posterior density of r, computed in Section 1:

$$p(r|k, K-1, D) = \frac{D}{B(k, K-k)} r^{Dk-1}(1-r^D)^{K-k-1} \tag{S.16}$$

We fill in $K = 2k$ to the previous expression:

$$p(r|k, 2k-1, D) = \frac{D}{B(k,k)} r^{Dk-1}(1-r^D)^{k-1} \tag{S.17}$$

The pdf of local dimension estimates δ can be expressed from the pdf of distances r with a simple intergal transform (change of variables):

$$p\left(r|k, 2k-1, D\right) \mathrm{d}r = q\left(\delta\right) \mathrm{d}\delta \tag{S.18}$$

so

$$q\left(\delta\right) = p\left(r|k, 2k-1, D\right) \left|\frac{\mathrm{d}r}{\mathrm{d}\delta}\right| \tag{S.19}$$

To compute the above expression, we first express r as a function of δ, then we compute the derivative. Afterwards we put the things together.

$$\delta = -\frac{\log 2}{\log r} \implies r = \exp\left(-\frac{\log 2}{\delta}\right) \implies \frac{\mathrm{d}r}{\mathrm{d}\delta} = \exp\left(-\frac{\log 2}{\delta}\right)\frac{\log 2}{\delta^2} \tag{S.20}$$

And finally, we put together these parts to get the pdf of the FSA estimator:

$$\begin{aligned} q(\delta|k, D) &= \frac{D}{B(k,k)} e^{\left(-\frac{\log 2}{\delta}(Dk-1)\right)} \left(1 - e^{\left(-\frac{\log 2}{\delta} D\right)}\right)^{k-1} e^{\left(-\frac{\log 2}{\delta}\right)} \frac{\log 2}{\delta^2} = \\ &= \boxed{\frac{D \log(2)}{B(k,k)} \frac{2^{-\frac{Dk}{\delta}}\left(1 - 2^{-\frac{D}{\delta}}\right)^{k-1}}{\delta^2}} \end{aligned} \tag{S.21}$$

where $B(k,k) = \frac{\Gamma(k)\Gamma(k)}{\Gamma(2k)}$ is the Euler beta function.

C Intuitive derivation for the asymptotic probability density of the median

For an odd sample size one can compute the asymptotic distribution of the sample median in an intuitive manner, if the distributions are given.

Let X be a continuous random variable and q and Q are it's probability density function and probability distribution respectively. Also, let $\{x_i\}_{i=1}^{n}$ be a sample of $n = 2l + 1$ odd sample-points drawn independently from the q density. We would like to derive the probability that the median falls into an infinitesimally small interval $\mathrm{d}x$ around $X = x$ value. The probability that the value of a sample point is smaller than x is given by $Q(x)$. Similarly the probability that the value is bigger than x is $1-Q(x)$. Also, the probability that a point exactly falls into a small range around x is given by $q(x)\mathrm{d}x$ by definition. For the whole sample $l = \frac{n-1}{2}$ points has to bigger and smaller than x and one sample has to be around it, so the probability is given by the following trinomial formula for an indepependent identically distributed sample:

$$P(x) = \frac{n!}{l!l!}\, Q(x)^l\, \left[1 - Q(x)\right]^l\, q(x)\,\mathrm{d}x \tag{S.22}$$

where P(x) is the probability that the median is at $X = x$ value. $Q(x)^l$ is the probability that l points has lower value than x and similarly $[1 - Q(x)]^l$ is the probability of l points has bigger value than x. The $\frac{n!}{l!l!} = \frac{1}{\mathrm{B}(l+1,l+1)}$ multiplier is a combinatorial normalizing constant, which can be written alternatively as an Euler-beta function with $l + 1$ as both of it's argument.

D Standard error of the median with Stirling approximation

According to Laplace, the probability distribution of the median is approximately Gaussian, with mean as the median (asymptotically) and with the variance:

$$\sigma^2 = \frac{1}{4nq(D)^2} \tag{S.23}$$

where n is the samples size and q is the pdf from which the sample was independently generated and D is the median.

We substitute eq. S.21 into eq. S.23 and by taking the squareroot, we get:

$$\sigma = \frac{2^{2k-2}\mathrm{B}(k,k)D}{\log(2)\sqrt{n}} \tag{S.24}$$

Using the Stirling approximation for the beta function ($k \to \infty$) we can simplify the above expression in the function of neighborhood size.

$$\mathrm{B}(k,k) \approx \frac{\sqrt{2\pi}\,k^{k-0.5}\,k^{k-0.5}}{(2k)^{2k-0.5}} = \frac{\sqrt{\pi}\,2^{1-2k}}{\sqrt{k}} \tag{S.25}$$

If we substitute back eq. S.25 into eq. S.24 we get the following approximate expression for the standard error of median:

$$\sigma \approx \frac{\sqrt{\pi}}{2\log 2}\frac{D}{\sqrt{nk}} \tag{S.26}$$

This formula expresses that the standard error of the median is proportional to the intrinsic dimension and shrinks with the squareroot of the sample size (n) and neighborhood size(k).

E Supplemental Figures and tables

STable 1: **Used symbols with interpretation.**

k	-	the order of the neighbor (increasing order as the distance from the center rises)
$K-1$	-	number of points in the neighborhood
R	-	distance from center
r	-	normalized distance from center $r = R/R_K$ ($r \in [0,1]$)
η	-	local density-dependent factor, approximately independent of R
D	-	intrinsic dimensionality of the space where the points are.
δ	-	local intrinsic dimension estimate
d	-	global intrinsic dimension estimate
P	-	Probability, probability mass function
p or q	-	probability density function (pdf)
q	-	probability density function of the mFSA estimate estimate
Q	-	probability distribution of the mFSA estimate estimate
n	-	sample size
N	-	number of realizations
B	-	Euler beta function

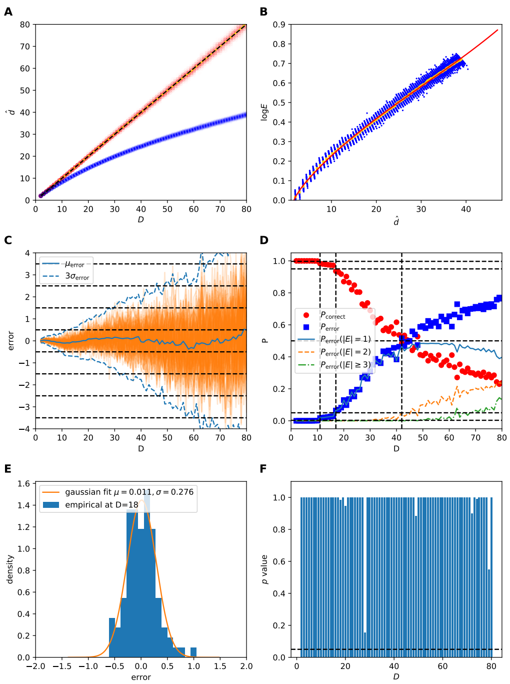

SFig. 1: **Calibration procedure for the** $n = 2500$ **datasets up to** $D = 80$ **(**$k = 5$**).** The figure shows the calibration procedure on 100 instances of uniformly sampled hypercubes. **A** Dimension estimates in the function of intrinsic dimensionality for the calibration hypercubes. The diagonal (dashed) is the ideal value, however the mFSA estimates (blue) show saturation because of finite sample and edge effects. cmFSA estimates (red) are also shown, with the mean (yellow) almost aligned with the diagonal. **B** The relative error (E) in the function of uncorrected mFSA dimension on semilogarithmic scale. The error-mFSA pairs (blue) lie on a short stripe for each intrinsic dimension value. The subplot also shows id-wise average points (yellow) and the polynomial fitting curve (red). **C** The error of cmFSA estimates in the function of intrinsic dimension on the calibration datasets. The mean error (blue line) oscillates around zero and the 99.7% confidence interval (blue dashed) widens as ID grows. The rounding switch-points are also shown. **D** The probability that cmFSA hits the real ID of data, or misses by one, two or more as a function of ID on the calibration dataset. **E** The error is approximately gaussian as shown through the empirical distribution at $D = 18$ with the fitted gaussian. **F** Results of normality test show, that the error do not deviate significantly ($alpha = 0.05$ dashed line) from a gaussian error distribution. We applied Bonferroni correction for multiple comparisons, the blue bars are the p-values.

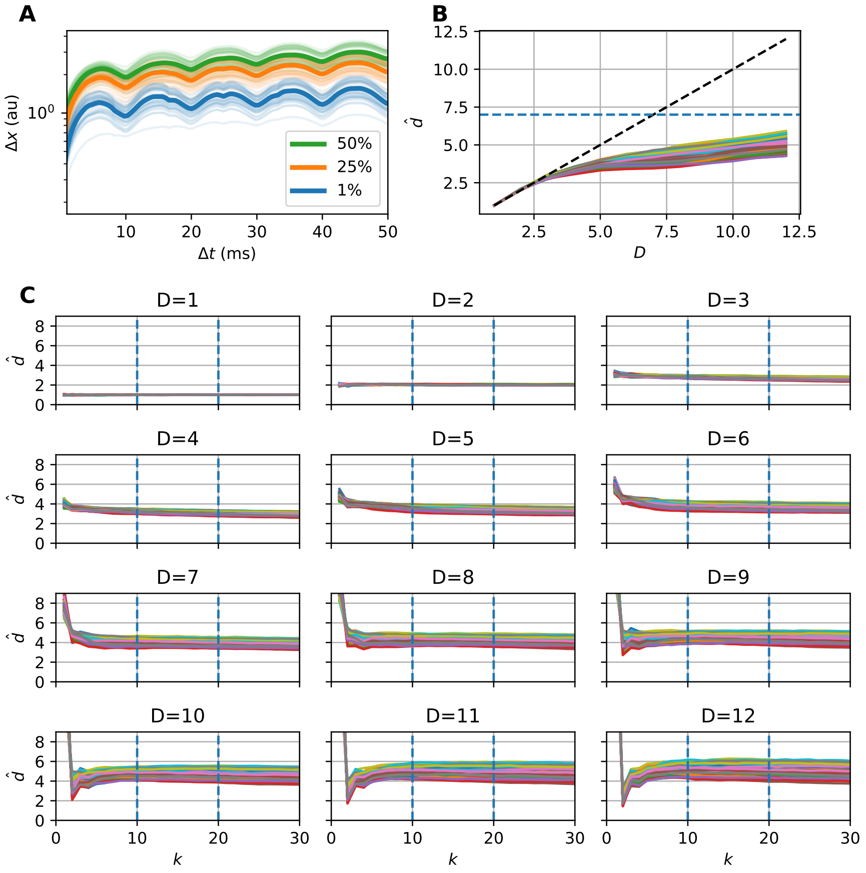

SFig. 2: **Subsampling and embedding of the CSD signals.** **A** Mean Space-time separation plot of the CSD recordings, the lines show the contours of the 1% (blue), 25% (orange), and 50% (green) percentiles for the 34 - 16 interictal and 18 seizures - recordings (thin lines) and their average (thick line, $D = 2$). The first local maximum is at around 5 ms (10 time steps), which appoints the proper subsampling to avoid the effect of temporal correlations during the dimension estimation. **B** Intrinsic dimension in the function of the embedding dimension for the 88 recording-channels (averaged between $k = 5 - 10$, for the first seizure). Dimension-estimates deviate from the diagonal above $D = 3$, thus we chose $D = 2*3+1 = 7$ as embedding dimension. **C** Intrinsic dimension in the function of neighborhood size for various embedding dimensions (88 channels, for the first seizure). The dimension estimates are settled at the neighborhood size between k=$10 - 20$ (dashed blue). The knee because of the autocorrelation becomes pronounced for $D >= 8$.
