# Supplementary figures and images for: Manifold-adaptive dimension estimation revisited
Source: PeerJ Comput Sci. 2022 Jan 6;8:e790. doi: 10.7717/peerj-cs.790 (PMC8771813; doi:10.7717/peerj-cs.790)

**A**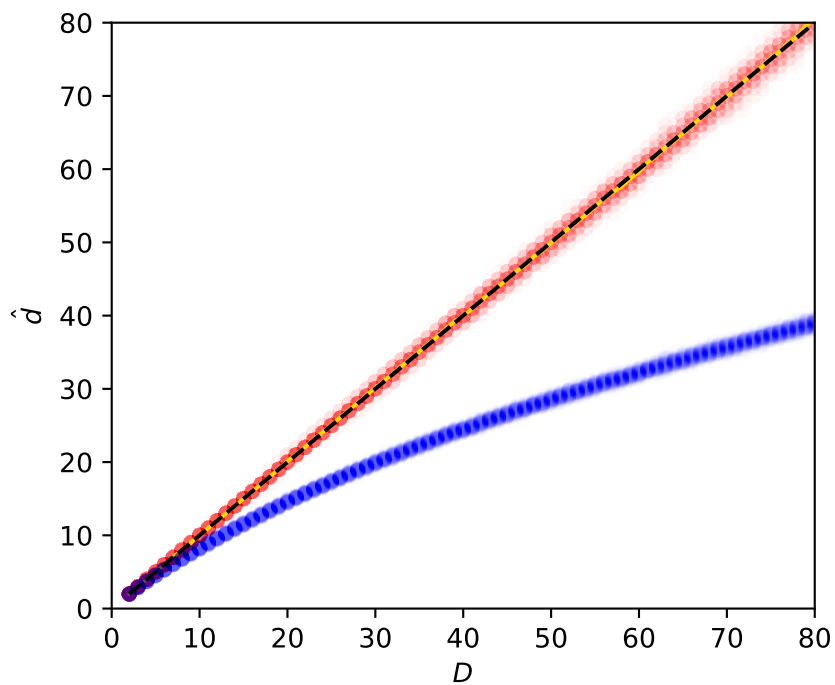**B**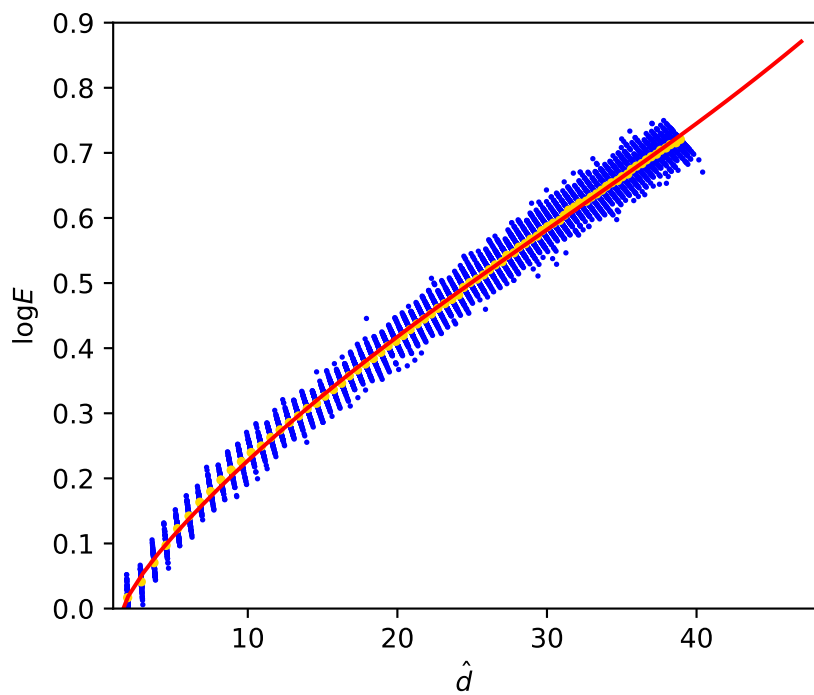**C**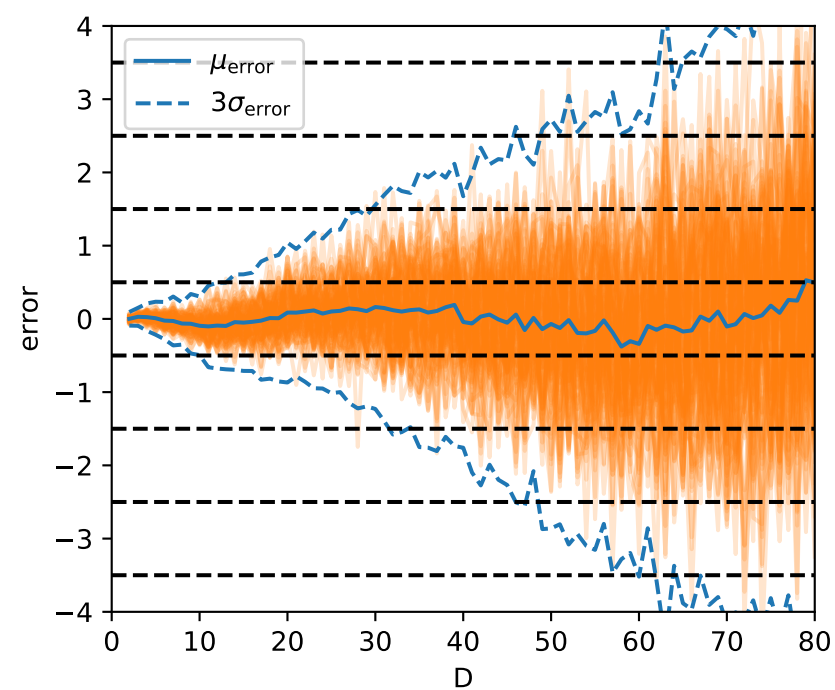**D**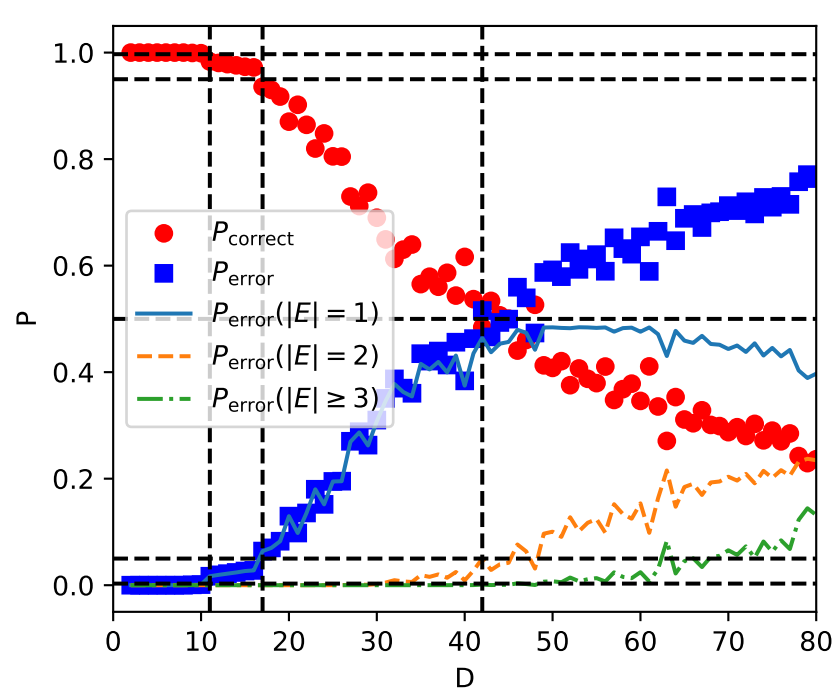**E**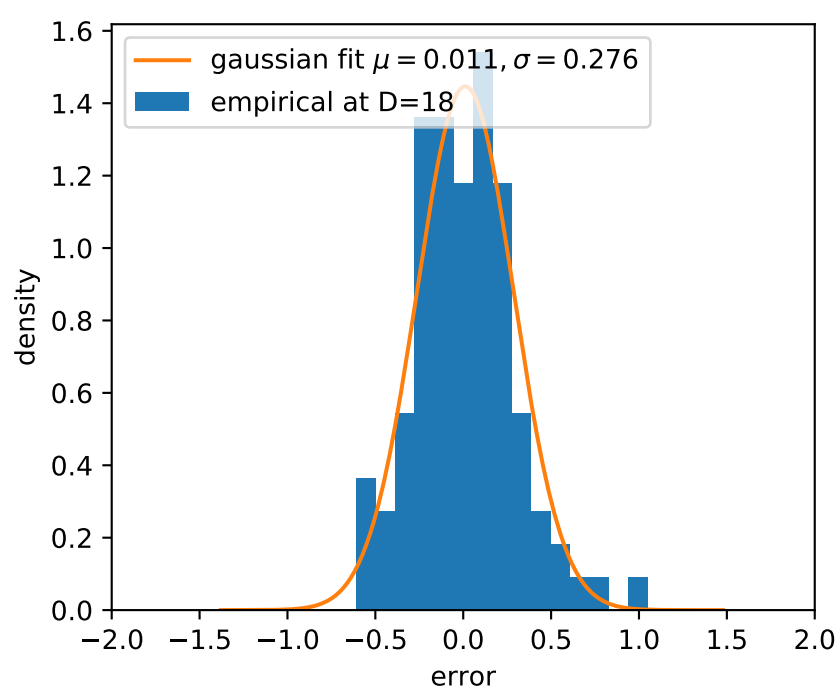**F**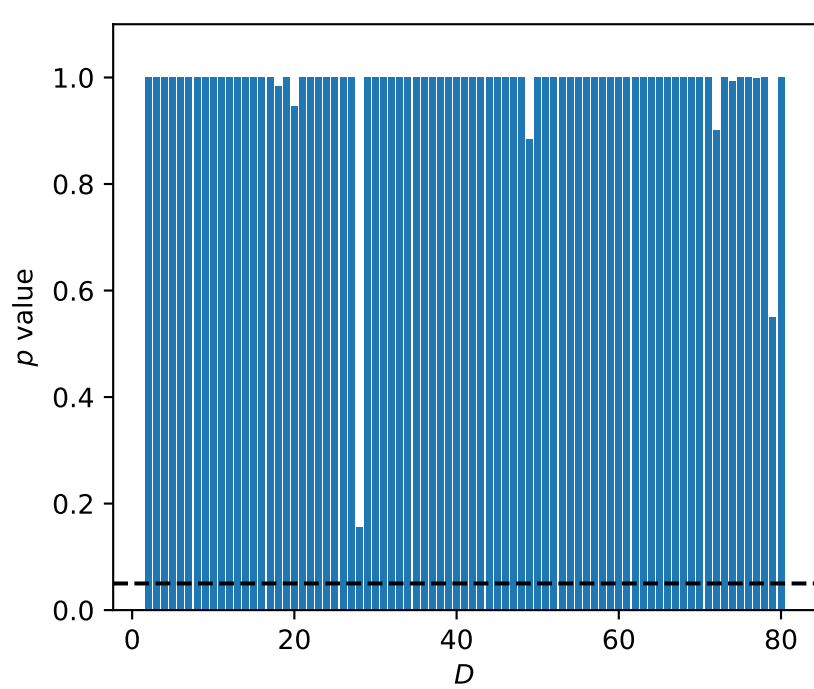

Supplement: Supplemental Information 2 — The figure shows the calibration procedure on 100 instances of uniformly sampled hypercubes. A Dimension estimates in the function of intrinsic dimensionality for the calibration hypercubes. The diagonal (dashed) is the ideal value, however the mFSA estimates (blue) show saturation because of finite sample and edge effects. cmFSA estimates (red) are also shown, with the mean (yellow) almost aligned with the diagonal. B The relative error (E) in the function of uncorrected mFSA dimension on semilogarithmic scale. The error-mFSA pairs (blue) lie on a short stripe for each intrinsic dimension value. The subplot also shows id-wise average points (yellow) and the polynomial fitting curve (red). C The error of cmFSA estimates in the function of intrinsic dimension on the calibration datasets. The mean error (blue line) oscillates around zero and the 99.7 confidence interval (blue dashed) widens as ID grows. The rounding switch-points are also shown. D The probability that cmFSA hits the real ID of data, or misses by one, two or more as a function of ID on the calibration dataset. E The error is approximately gaussian as shown through the empirical distribution at D = 18 with the fitted gaussian. F Results of normality test show, that the error do not deviate significantly (alpha =0.05 dashed line) from a gaussian error distribution. We applied Bonferroni correction for multiple comparisons, the blue bars are the p-values. [file peerj-cs-08-790-s002.pdf]

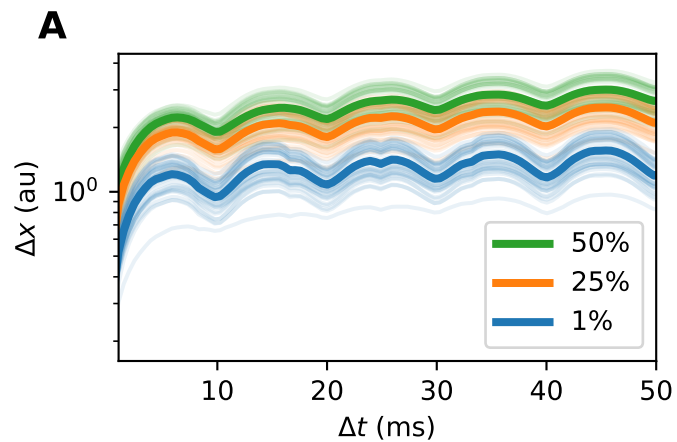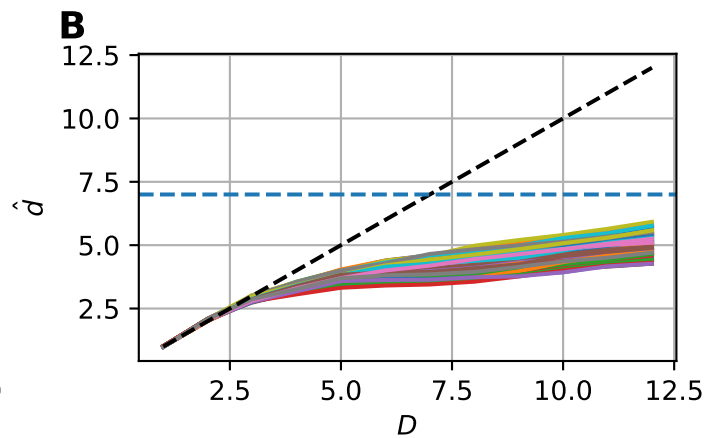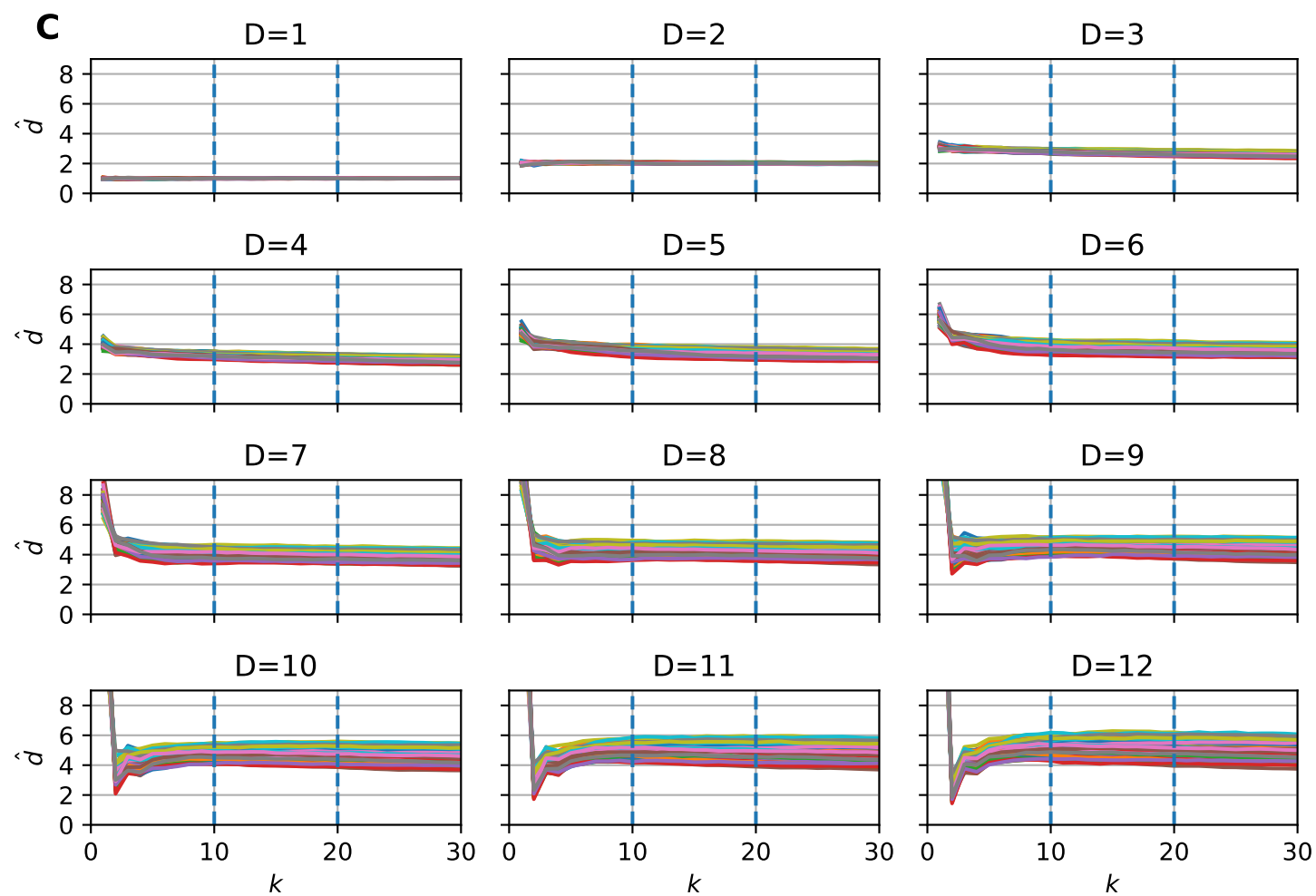

Supplement: Supplemental Information 3 — A Mean Space–time separation plot of the CSD recordings, the lines show the contours of the 1% (blue), 25% (orange), and 50% (green) percentiles for the 34–16 interictal and 18 seizures - recordings (thin lines) and their average (thick line, D = 2). The first local maximum is at around 5 ms (10 time steps), which appoints the proper subsampling to avoid the effect of temporal correlations during the dimension estimation. B Intrinsic dimension in the function of the embedding dimension for the 88 recording-channels (averaged between k =5–10, for the first seizure). Dimension-estimates deviate from the diagonal above D = 3, thus we chose D =2*3+1 =7 as embedding dimension. C Intrinsic dimension in the function of neighborhood size for various embedding dimensions (88 channels, for the first seizure). The dimension estimates are settled at the neighborhood size between k =10–20 (dashed blue). The knee because of the autocorrelation becomes pronounced for D≥8. [file peerj-cs-08-790-s003.pdf]
